# Computational study of the water-driven graphene wrinkle life-cycle towards applications in flexible electronics

**DOI:** 10.1038/s41598-020-68080-5

**Published:** 2020-07-09

**Authors:** Jatin Kashyap, Eui-Hyeok Yang, Dibakar Datta

**Affiliations:** 1 Department of Mechanical and Industrial Engineering, New Jersey Institute of Technology, Newark, NJ 07103 USA; 2 Department of Mechanical Engineering, Stevens Institute of Technology, Hoboken, NJ 07030 USA

**Keywords:** Electronic properties and devices, Mechanical and structural properties and devices, Atomistic models

## Abstract

The ubiquitous presence of wrinkles in two-dimensional materials alters their properties significantly. It is observed that during the growth process of graphene, water molecules, sourced from ambient humidity or transferred method used, can get diffused in between graphene and the substrate. The water diffusion causes/assists wrinkle formation in graphene, which influences its properties. The diffused water eventually dries, altering the geometrical parameters and properties of wrinkled graphene nanoribbons. Our study reveals that the initially distributed wrinkles tend to coalesce to form a localized wrinkle whose configuration depends on the initial wrinkle geometry and the quantity of the diffused water. The movement of the localized wrinkle is categorized into three modes—bending, buckling, and sliding. The sliding mode is characterized in terms of velocity as a function of diffused water quantity. Direct bandgap increases linearly with the initial angle except the highest angle considered (21°), which can be attributed to the electron tunneling effect observed in the orbital analysis. The system becomes stable with an increase in the initial angle of wrinkle as observed from the potential energy plots extracted from MD trajectories and confirmed with the DOS plot. The maximum stress generated is less than the plastic limit of the graphene.

## Introduction

Graphene like two dimensional (2D) materials are less resistant to buckling, and they tend to form surface corrugations such as wrinkles, ripples, crumples^1^. Depending upon applications, wrinkling can be “either boon or a bane”. The ubiety of the surface textures significantly alters the mechanical, electronic, and optical properties, which can be exploited in various applications in the fields of electronic^2^, sensing and actuation^3^, energy storage^4,5^, chemical reactions^6–8^, and bioelectronics^9^. Given the literature, applications in flexible electronics stand out among the others. Initially, wrinkles in graphene were detected during the synthesis processes^6,7,10–12^ Usually, amid the synthesis process of graphene, water molecules sourced from the ambient air/transfer process can get diffused in the system. The diffused water can significantly influence the geometrical parameters of the wrinkles, and consequently, the properties of the graphene^8^ in addition to the substrate effect^13^. There are other ways for the water to get diffused in the system. For example, Raghav et al. had studied the wedging of a graphene bilayer by water molecules^14^. The first on-purpose fabrication of the wrinkles was demonstrated in 2011^15^, followed by multiple studies on modeling the wrinkle theoretically/numerically^11^^,^^16,17^.

Deformable Thin Film Transistors (TFTs) are core components in flexible electronics. Major obstacles in developing flexible TFTs are fragility and the current leakage. Surface instabilities in thin-film TFTs are exploited to overcome these shortcomings. A Single-Walled Carbon Nanotube(SWCNT)/wrinkled Al_2_O_3_ TFT displayed minimum current leakage of 10^−13^ A, attributed to the airgaps present underneath the wrinkles^12^. Additionally, the In–Ga–Zn–O (IGZO), pentacene, reduced graphene oxide(rGO), and rGO/ Polydimethylsiloxane(PDMS) based TFTs are observed to preserve the electrical performance and mechanical stability under the strain of 20% due to wrinkles’ existence^18–21^. Similar behavior was observed in silver, and rGO based interconnects^17,18^^,^^22^ It inspired the investigation of dependency of the device performance on individual geometrical parameters of the wrinkles. In an IGZO TFT and rGO interconnect, the wrinkles’ wavelength and amplitude were controlled in a way so the device can be bent up to 13 μm and strained up to 5% while preserving the electrical performance^17^^,^^23,24^. Nirmalraj et al.^25^ had demonstrated controlling the Pentacene growth on a MoS_2_ substrate by tuning the wrinkle height. Furthermore, the non-uniformity of the wrinkled 2D materials’ topology can be used for photon trapping in between trough and vertex. This lead to the design of a wearable laser device with a threshold below than ever reported^26^. TFTs are usually decorated with mechanical sensors. A crumpled rGO based piezoresistive pressure sensor developed high stretchability, strain insensitive resistance profile, and increased sensitivity^27^. In another study, a wrinkled rGO sheet improved the sensitivity and range at non-stretched state too^28^. In addition to pressure and position detection, strain measurements are equally important in flexible electronics. Wrinkle assisted crack structures can be used to design strain sensors^29^. While subjected to bending stresses, the resulting sensors showed high sensitivity, high stretching range, low limit of detection, high durability, wider strain range. A wrinkled 2D complex-based strain sensor is demonstrated to acquire a high gauge factor of 1071^30^ and sustain the electrical signals over a wide range of deformation^15^. The intrinsic piezoresistive property and electron tunneling effect (observed in our work in Fig. 11d) in a graphene crystal were attributed to high sensitivity, wide range, and stretchability^30^. Additionally, the effect of engineering the wrinkle pattern in tuning adhesion, wetting, open‐channel microfluidics, and responsive microlens arrays is studied experimentally^31^.

**Figure 1:**
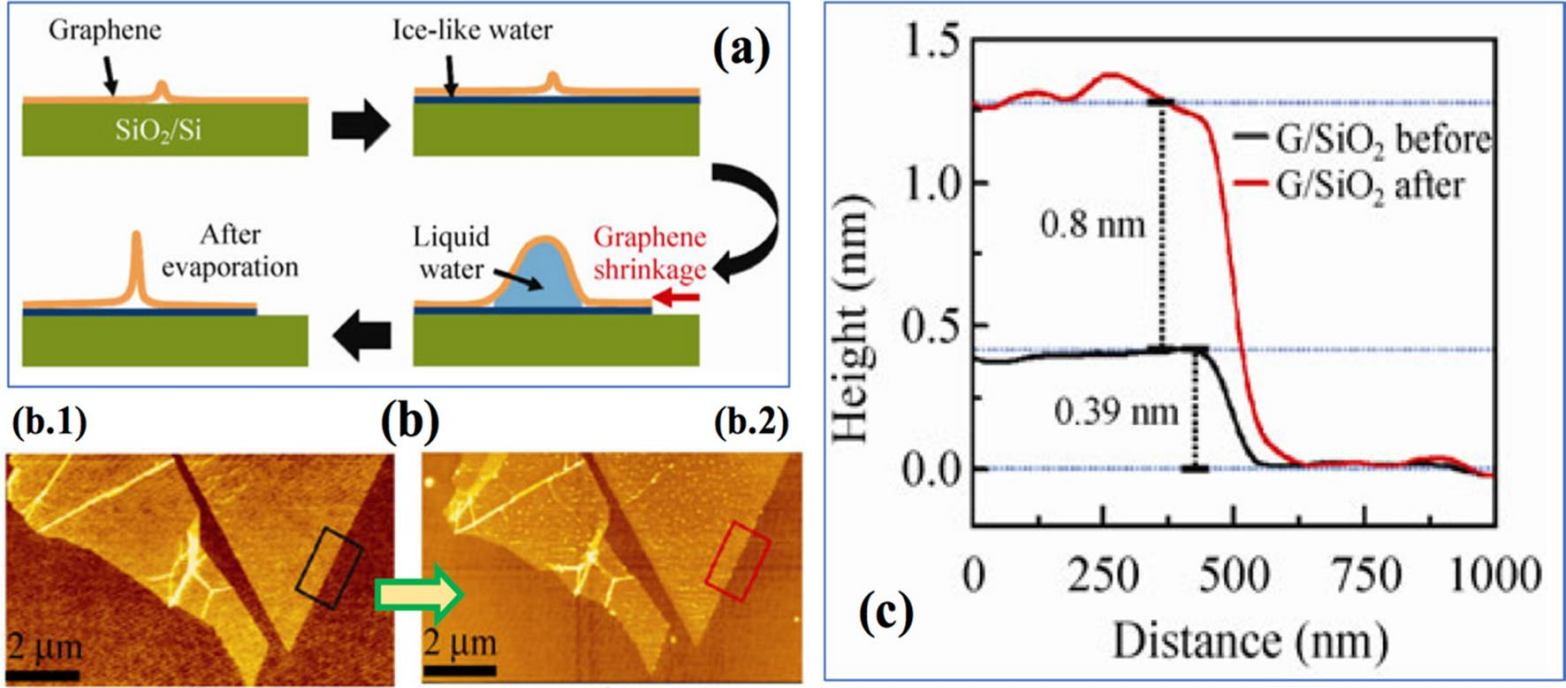
(**a**) Schematic diagram of the wrinkle formation process through the ice-like water formation and liquid water diffusion at high humidity, and liquid water evaporation in a dry environment. (**b**) Height changes in a graphene/SiO_2_ structure after exposure to high humidity. AFM topographic images of a monolayer graphene mechanically exfoliated on SiO_2_ (b.1) before and (b.2) after the high humidity exposure. (**c**) Averaged height profiles measured for the areas marked with black and red rectangle in (b.1) and (b.2), respectively. G denotes monolayer graphene. © Copyright : Springer Science + Business Media, Lee et al.^40^.

The presence of wrinkles in different 2D systems, including Co/Cu/PDMS, has attributed to preserving Giant Magnetoresistance performance(GMR) even at strains as high as 4.5%^32,33^. The wrinkles’ patterns were observed to affect GMR and magnetic field sensitivity uniquely over a wide range of strain^34^. Often, there are sensitive parts of the device which should be shielded against the magnetic fields. A wrinkled polyurethane(PU)/MXene(Ti_3_C_2_T_x_) complex was tested for its electromagnetic wave interference(EMI) capability in flexible electronics^35^. The power storage components are also needed to be able to withstand mechanical deformations. Wrinkled/crumpled supercapacitors based upon graphene/rGO are demonstrated to have high capacitance at strains up to 300% and 5,000 stretching cycles^36,37^. Further, wrinkled 2D material based bioelectrodes seem to show no degradation in charge capacity and impedance while used for in-vivo measurements^38^. Wrinkles in Polyimide/Silbione system has resulted in low elastic modulus and high tangent modulus to avoid fracture and mechanical constraint on the movement of a biological tissue over a substrate, respectively^39^. Crumples in a 2D material can undergo a geometrical transformation for various reasons. A majority of systems acquired the same type of wrinkle as one can observe while growing graphene on a SiO_2_ substrate. A related work by Lee et al.^40^ revealed that during the transfer of exfoliated graphene onto a SiO_2_ substrate, water molecules get diffuse between graphene and substrate (Fig. 1). The diffused water and subsequently its evaporation cause wrinkles and change in geometry of the wrinkles, respectively, resulting in Wrinkled Graphene Nanoribbon (WGNR). It is essential to study wrinkle transformation lead by water diffusion and evaporation in the system.

In this work, we provided a detailed explanation of water diffusion and its impact on a wrinkled bilayer graphene system characterized by geometrical parameters and electronic properties. Our results can explain the phenomenon of the water interaction with the graphene systems^36,37^, and change in electronic properties of different 2D materials caused by wrinkles^38,40^. Our study is composed of three phases. Phase I addresses the wrinkle evolution due to diffused water in between graphene with distributed wrinkles and a graphene substrate. We had varied the amount of diffused water and the initial angle of the distributed wrinkles in graphene and studied the formation of final localized wrinkle configuration and the stress generated in wrinkled graphene. Phase II deals with the evaporation of diffused water, and the effect of evaporation on the collapsing of the wrinkle, i.e. completion of wrinkle life-cycle. We observed that, upon complete evaporation, the resulting localized wrinkle undergoes motion that is composed of three fundamental modes—bending, buckling, and sliding as studied theoretically by Im et al.^41^ Lastly, Phase III uses Density Functional Theory (DFT) while considering the free-standing WGNR with four different initial angles and studies its influence on the electronic properties. Only quasi-periodic wrinkles are considered instead of a complex network of wrinkles that exist in the experimental setups. However, we believe that our studies will encourage researchers to study more complicated wrinkle structures.

## Models and methods

### Molecular dynamics (MD)

**Figure 2:**
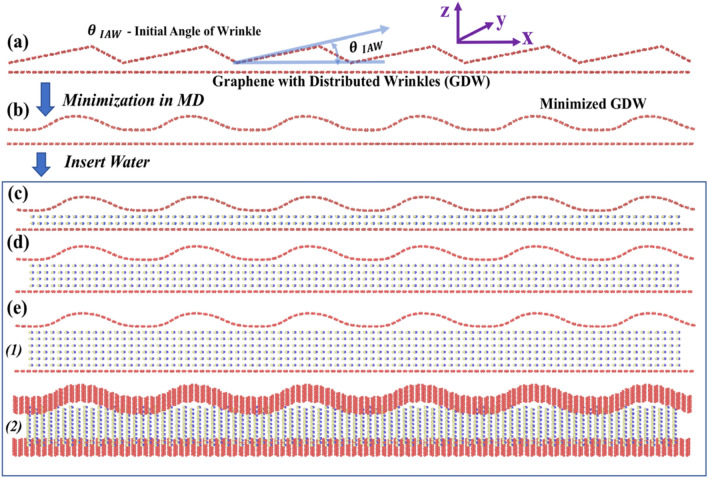
(**a**) V-shaped, hypothetical, wrinkling structure considered for the study (generated using Matlab code). Different Initial Angle of Wrinkling (IAW), ${\theta \, }_{IAW }=$ 6°, 11°, 16°, 21°, are considered in this study. (**b**) Minimization of the system by MD (no water case). (**c**–**e**) Insertion of water for further studies. (**c**) Two- (**d**) four-, and (**e**) six-layer of water. (e.1) front view and (e.2) inclined view.

This study is divided into *three phases* based on which part of the wrinkle life-cycle is being studied, i.e., formation, evolution, collapse, and the approach used for analysis, i.e., MD or DFT. In the *first phase*, we investigated the wrinkle formation and evolution. In this phase, as shown in Fig. 2a, using the in-house MATLAB code, we created a Graphene with Distributed Wrinkles (GDW) supported by a Flat Graphene (FG) with Periodic Boundary Conditions (PBC) (width = 20 $\AA$). Here, we considered different Initial Angle of Wrinkle (IAW) denoted as ${\theta }_{IAW}$ (${\theta }_{IAW}$ = 6°, 11°, 16°, 21°) to generate GDW with four different configurations. Figure 2a shows a representative case of ${\theta }_{IAW}= 11^\circ$. While changing ${\theta }_{IAW}$, we kept the number of carbon (C) atoms in GDW the same, but the size of the FG is reduced. For example, if we take a flat graphene sheet and keep compressing to get wrinkles of different angles, the number of atoms in the sheet remains unchanged, but the overall length of the sheet is reduced. Table S1 of the Supplementary Information (SI) shows the C atoms for different ${\theta }_{IAW}$. We define a parameter called Carbon Ratio (CR), which is the ratio of the number of C atoms in GDW to that of FG. For ${\theta }_{IAW}$ of 6°, 11°, 16°, 21°; CR values are 1.017, 1.053, 1.11, 1.20, respectively (Table S1).

The Adaptive Intermolecular Reactive Bond Order (AIREBO) potential was used for the C–C interactions^42^. We performed an energy minimization of this system (Fig. 2a) using the conjugate gradient method, as implemented in the MD package: LAMMPS^43^. Figure 2b shows the minimized/equilibrium configuration of GDW supported by the FG. At this point, we studied wrinkling evolution for four cases—(a) without water (Fig. 2b), and (b) two-, (c) four-, and (d) six-layer of water in between the GDW and FG (Fig. 2c–e). In-house MATLAB code was used to insert the water layer in the wrinkled system shown in Fig. 2b. The density of the added water molecules, i.e., the number of water molecules per unit volume, is the same for all cases. Table S2 of SI shows the number of water molecules for different cases.

The water molecules were added to the system (Fig. 2b) in two different ways—(1) While adding the water, the gap between the graphene layers was increased to maintain a fixed distance between the water layers and the adjacent graphene sheet (Figs. 2c–e). (2) Water layers were added while keeping the distance between GDW and FG to a fixed distance corresponding to the maximum water case considered (i.e., gap shown in Fig. 2e is considered for all other cases). However, in both cases, we qualitatively observed the same results. Therefore, we only discuss the results for the cases shown in Figs. 2b–e. We used TIP4P potential for water (H_2_O)^44^, and Lennard–Jones type of pair potential for C and H_2_O interactions^45^. We used TIP4P potential before for water modeling^3,37^, and our computational results were in good agreement with the experimental findings^3^.

**Figure 3:**
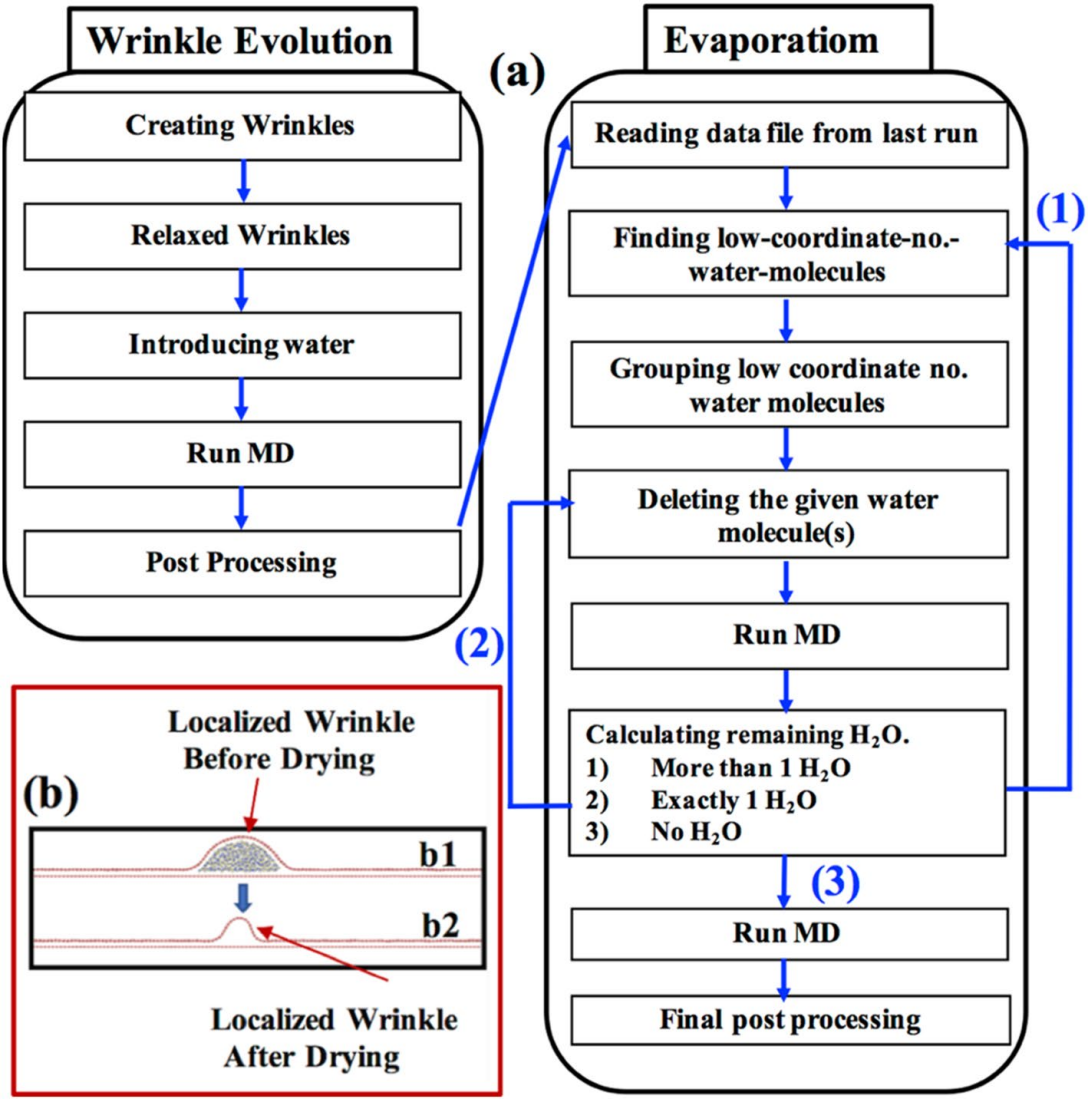
(**a**) Flowchart showing the algorithm used for the Molecular Dynamics (MD) simulation for wrinkle evolution and water evaporation. (**b**) Snapshot of complete water evaporation.

The methodology of *Phase I* is summarized in the ‘Wrinkle Evolution’ part of the flow-chart shown in Fig. 3. The temperature of the system was controlled at 300 K using the Berendsen thermostat^46^. We then performed MD simulation using NVE ensemble with the timestep of 1 femtosecond. Sufficient MD steps (at least one million steps) were performed to make sure the system reached its lowest energy configurations (discussed later in detail). After the end of the first phase simulations, we stored the system configuration and reused it as the initial structure for the next phase of the project, i.e., water drying case. We performed the stress analysis of the final stabilized graphene sheet with a localized wrinkle using the in-built stress computation algorithm of LAMMPS^47^. The detail of the stress calculation methods is provided in the supplementary information (section D).

In *Phase II*, we studied how wrinkle collapses due to the drying of water. The ‘Evaporation’ part of Fig. 3 summarizes the methodology for studying the drying phase. For drying modeling, we started with the last configuration of the system in the first phase, e.g., schematic in Fig. 3b1. In our LAMMPS script, the water molecules at the outer periphery were identified. The identification process was accomplished by locating and then grouping those water molecules having lesser coordination numbers as compared to their counterparts inside the water-body. The identified surface water molecules were deleted, followed by running the MD simulation for 2000 steps. The same loop was performed again (water deletion and MD simulation) until all water molecules were deleted.

### Density functional theory (DFT)

In *Phase III,* the DFT based analyses were performed on one frame from each case considered from Phase I, as shown in Figs. 11, 12, 13. In this phase, we tried to understand the impact of geometrical parameters of WGNR on their fundamental electronic properties. We implemented first-principles DFT with plane-wave basis sets and pseudopotentials to describe the electron–ion interactions as implemented in Quantum Espresso (QE)^48^. All calculations were done using the projector augmented wave (PAW) pseudopotentials and the Perdew–Burke–Ernzerhof (PBE) exchange–correlation functional^49^. The plane-wave basis sets were used with a plane-wave cutoff energy of 100 eV, and a kinetic energy cutoff for augmentation charges of 400 eV. The convergence threshold of Kohn–Sham equations was set to 1*e*−06. The gamma-centered *k*-point sampling grids obtained using the Monkhorst–Pack method, were 8 × 8 × 8 with a unit offset for the graphene unit cell. The valence electrons contain *s* and *p* orbitals for carbon. Before DFT calculations, all atoms in the cell, as well as the lattice dimensions and angles, were relaxed to the equilibrium configurations by using MD. For consistency, only one case corresponding to two water (H_2_O) layers was studied from each considered IAW, i.e., 6°, 11°, 16°, 21° (Fig. 11). Furthermore, the systems from all four cases were trimmed down to 120 atoms to eliminate the system size dependency, and conform to the available computational resources. For the band structure calculations, the symbols and coordinates of the high-symmetry points in the first Brillouin zone of the crystals were taken from Y Hinuma et al.^50^. MATLAB and VESTA codes were used for the post-processing of the results.

## Results and discussions

We discuss the results for three phases—(I) The formation and evolution of wrinkles for no-water, and water inside GDW and supported FG (without drying), (II) The collapse of the localized wrinkle during water drying, and (III) The electronic structure of free-standing WGNR for four different geometries.

### Phase I: The formation and evolution of wrinkles due to diffused water

**Figure 4:**
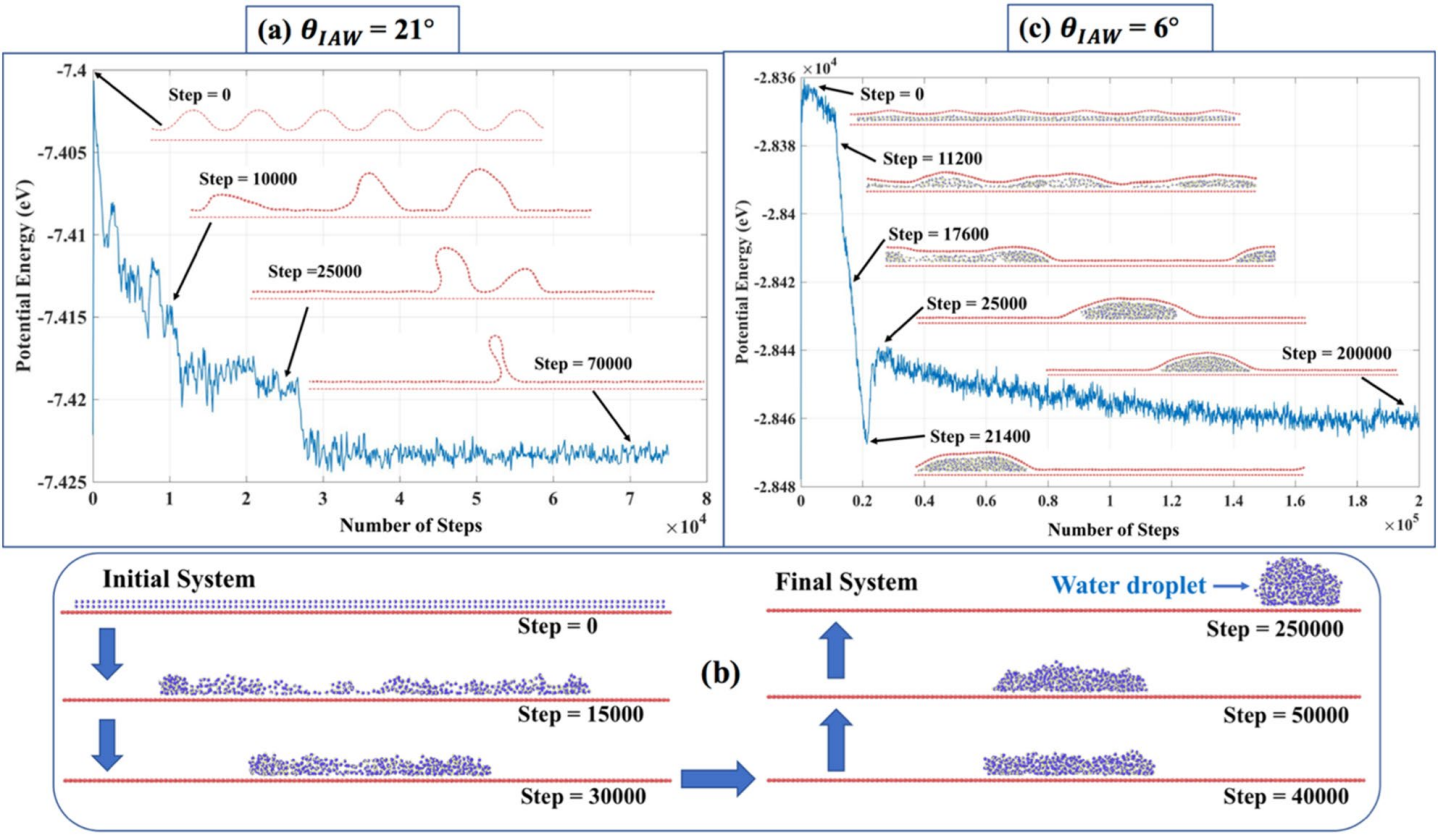
(**a**) Potential energy variation during the wrinkle amalgamation for ${\theta }_{IAW}$ = 21° (no water case), (**b**) formation of the water droplet on graphene, (**c**) Potential energy variation during wrinkle amalgamation (${\theta }_{IAW}$ = 6° for two water layer case.)

As discussed in “Introduction” section, during the synthesis^51^ and/or transfer process^52^, graphene can inherent distributed wrinkles, and they tend to coalescence to form a localized structures, i.e., from ‘Graphene with Distributed Wrinkles (GDW)’ to ‘Graphene with Localized Wrinkle (GLW)’. For the simplicity of analysis, we considered here GDW only among other available morphologies (Fig. 2b), i.e., ripples, crumples, folds, etc. First, we analyzed how GDW transforms into GLW when there is no diffused water (Fig. 2b). Figure 4a shows the case of distributed wrinkles for the representative case of ${\theta }_{IAW}=21^\circ$ and CR = 1.21. As mentioned earlier in “Models and methods” section, Carbon Ratio (CR) is the ratio of C atoms in the upper sheet with wrinkles and the underlying flat sheet (see Table S1 of SI). The video related to this phenomenon is shown in the SI (Movie_Figure4a.mp4). The distributed wrinkles on same-side amalgamate to form a localized wrinkle, i.e., GDW transforms to GLW. In crystalline solids, same-sign dislocations repel according to Frank’s rule^53^. However, in van der Waals (vdW) layered structures, such as graphene, same-sign (same-side) winkles attract each other to form a localized wrinkle. A similar observation was reported for the MoS_2_ bilayer system^54^. As shown in Fig. 4a, for ${\theta }_{IAW}=21^\circ$, first, the initially distributed wrinkles transform into intermediate wrinkle localization and finally single ‘localized wrinkle’. During the intermediate steps (step = 10,000, 25,000), initially distributed wrinkles form three followed by two wrinkles and, finally, one ‘localized wrinkle’. There is a drop in potential energy during the transformation of three to two and, finally, one wrinkle. In SI, Figures S1, S2, S3 show the evolution of initial wrinkle for three other cases—(1) ${\theta }_{IAW}=6^\circ$, CR = 1.017; (2) ${\theta }_{IAW}=11^\circ$, CR = 1.053; (3) ${\theta }_{IAW}=16^\circ$, CR = 1.11. As shown in Figure S1, when the ${\theta }_{IAW}$ is less (e.g., ${\theta }_{IAW}=6^\circ$), and CR is close to 1, initially distributed wrinkles do not converge to single localized wrinkle, but tries to transform into a highly strained flat sheet.

Figure 4b represents a case-study where water molecules are initially placed on monolayer FG with no graphene sheet on top. The SI contains the related video (Movie_Figure4b.mp4). The MD simulation shows that water molecules coalescence to form a water droplet because of the hydrophobicity of graphene^55^ with a contact angle of (≈)125°, which matches with the literature^56^. In Fig. 4c, we considered a representative case of ${\theta }_{IAW}=6^\circ$ (two-layer water) to discuss how diffused water molecules inside GDW and FG influence the wrinkle evolution. The SI contains the related video (Movie_Figure4c.mp4). The presence of diffused water causes ‘competition’ between two phenomena—(i) wrinkle amalgamation, and (ii) droplet formation. The final localized wrinkle configuration is primarily determined by the dominant phenomenon. Figure S1 shows that for GDW with ${\theta }_{IAW}=6^\circ$, initial wrinkles tend to merge together to form a flat sheet. Figure 4b shows the water droplet formation starting from the initial water layer. When these phenomena, i.e., wrinkling formation and droplet formation interact with each other, the droplet formation process dominates. However, the final droplet shape in this case (Fig. 4c) is not the same as in Fig. 4b. Because in this case, the upper graphene sheet tries to compress the water droplet, as observed by McKenzie et al.^57^. However, since the droplet formation phenomenon is dominant here, the final wrinkle configuration is primarily determined by the water droplet.

**Figure 5:**
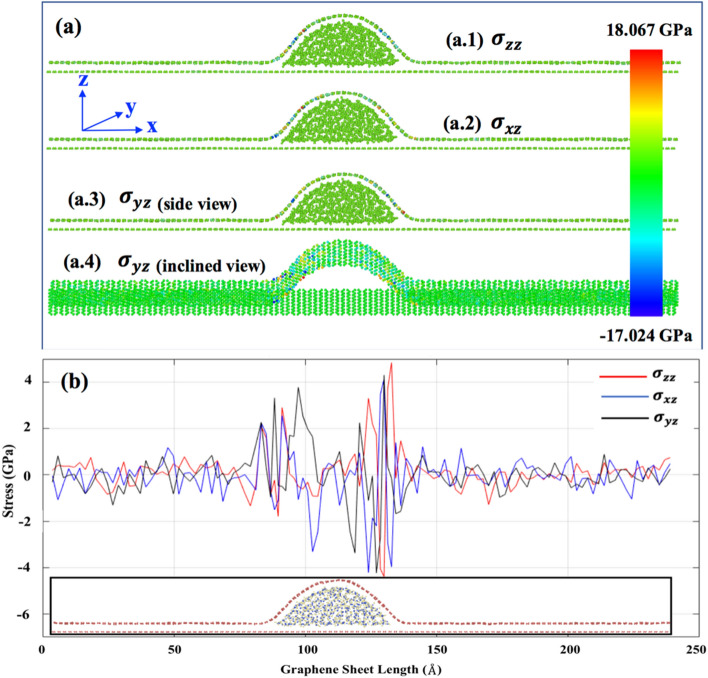
(**a**) Cauchy stress field distribution in the localized wrinkle graphene system for a particular time frame—[a.1] ${\sigma }_{zz}, [$a.2] ${\sigma }_{xz}$, [a.3]—${\sigma }_{yz}$side view, [a.4] ${\sigma }_{yz}$—inclined view, (**b**) Stress distribution across the top wrinkled graphene sheet. Stresses are averaged upon the whole line of atoms along the y-axis.

Figures S4, S5, S6, S7 show the final wrinkle configuration of graphene starting from the initial wrinkle angle of ${\theta }_{IAW}=6^\circ$, $11^\circ$, $16^\circ$, and $21^\circ$ respectively for 0-, 2-, 4-, and 6-layer water cases. The stress generated during the wrinkle formation is shown here. We considered a representative case of ${\theta }_{IAW}=11^\circ$ for the 2-layer water case (Figure S6b) for analyzing the stress generation during the wrinkle formation. Figure 5 shows the *z*-component of the Cauchy stress. Considering the stress-field in Fig. 5a, we note that atoms in the flat regions and at the top of the wrinkle have no *z*-component stress. Only the sidewalls, i.e., the base of the localized wrinkle, are stressed. Figure 5b shows the distribution of the *z*-component of stress across the GLW (Graphene with Localized Wrinkle). Here, stresses are averaged upon the whole line of atoms along the periodic *y*-axis. We note that for all three stress cases (${\sigma }_{zz}, {\sigma }_{xz},{\sigma }_{yz}$), the stress is high in the sidewalls (the base) of the wrinkle as expected^58^. The maximum individual atomic stress, observed in the wrinkle, varies from around − 17 GPa (compressive) to 18 GPa (tensile) (Fig. 5a). When averaged over the stripe of atoms along the periodic *y*-axis, the maximum magnitude of stress is around 4 GPa (Fig. 5b). In summary, stress generation in graphene during the wrinkle formation is much lower than the stress that can cause plastic deformation^59,60^. Hence, water diffusion-induced wrinkle formation in graphene does not cause in-plane lattice-strain high enough to break the bonds and cause plasticity.

**Figure 6:**
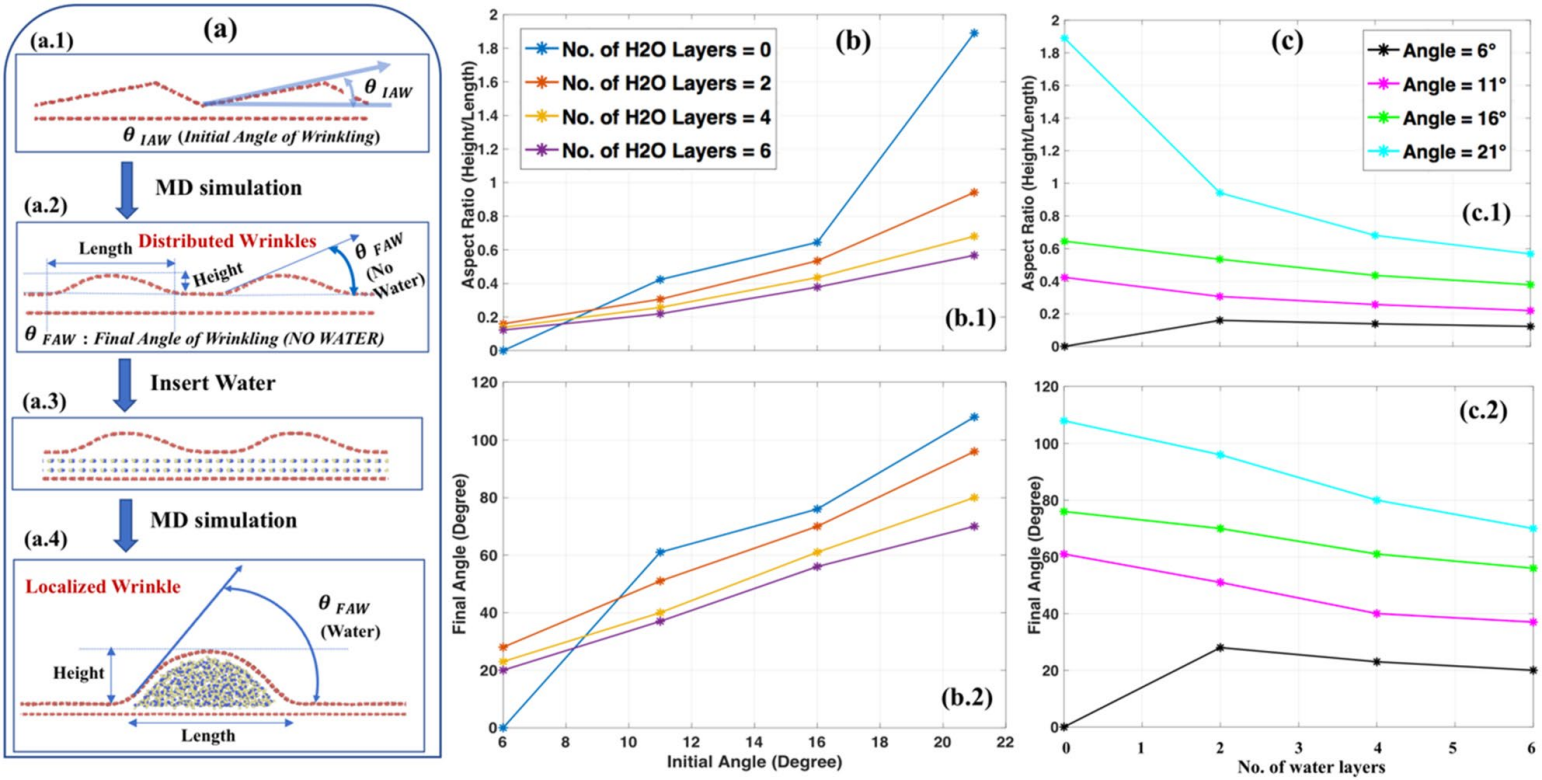
(**a**) Wrinkle configurations at different stages.[a.1] Model considered at the beginning of the simulation showing the ‘Initial Angle of Wrinkle (${\theta }_{IAW}$)’.[a.2] Wrinkle configuration obtained after MD simulation of Figure a.1 with specific height, length, and Final Angle of Water ${(\theta }_{FAW})$ of the wrinkled structure (with no water). [a.3] Insertion of water molecules in the minimized wrinkled structure obtained in Figure a.2. [a.4] Final configuration (after MD simulation of Figure a.3) with final height, length, and ${\theta }_{FAW}$ of the wrinkled structure (with water). (**b**) Variation of the [b.1] aspect ratio (height/length), and [b.2] final angle (${\theta }_{FAW}$) of localized wrinkle obtained from different initial angles (${\theta }_{IAW}$). At each ${\theta }_{IAW}$, we varied the number of water molecules inside the graphene bilayer.[c] The aspect ratio (AR) and final angle variation w.r.t. the no. of water layers.

As shown in Figures S5, S6, S7,S8 irrespective of the number of the diffused water layers (i.e., 2-, 4-, or 6-layer), the pattern of the final configuration is the same for all water layer cases. We define two new parameters, as shown in Fig. 6a—(1) *Aspect Ratio (AR)* as the ratio of the height of the wrinkle to its base length, and (2) ‘*Final Angle of Wrinkle*, ${\theta }_{FAW}$’. If *AR* = *0,* it means the sheet is flat (height = 0). The higher the value of *AR,* the lesser the radius of the curvature of wrinkle-vertex. Moreover, there is a directly proportional relationship between AR and ${\theta }_{FAW}$. Figure 6b,c show how *AR* and ${\theta }_{FAW}$ vary with ${\theta }_{IAW}$ (*initial angle of water*) for the differing number of the water layers. When there is no diffused water (Figures S5a, S6a, S7a, S8a), as ${\theta }_{IAW}$ increases from $6^\circ$ to $21^\circ$, the final localized wrinkle becomes sharper. Therefore, the blue curve (*no water case*) in Fig. 6b shows that the *AR* and ${\theta }_{FAW}$ increase as ${\theta }_{IAW}$ increases. As mentioned before, the presence of diffused water starts the complex interplay between the wrinkle amalgamation and droplet formation^61^. The final shape is the result of the interaction between two phenomena. For 2-, 4-, or 6-layer diffused water cases (Figures S5b–d, S6b–d, S7b–d, S8b–d), *AR* and ${\theta }_{FAW}$ linearly increases as ${\theta }_{IAW}$ increases from $6^\circ$ to $21^\circ$. As shown in Figure S5a, for ${\theta }_{IAW}= 6^\circ$ case, the graphene sheet with initially distributed wrinkle flattens (*AR* = *0, *${\theta }_{FAW}= 0^\circ$ in Fig. 6b.1) for no water case. With the insertion of 2-, 4-, or 6-layer diffused water (Figure S5b-d), the droplet and wrinkle size increases. However, the decrease in *AR* for a different water-layer case for ${\theta }_{IAW}= 6^\circ$ is not significant (*black* line in Fig. 6c.1) as the length and height of the wrinkle increase almost proportionately. For the ${\theta }_{IAW}= 21^\circ$ case (Figure S8), we have the spiker localized wrinkle for no-water case (Figure S8a). The *AR*, in this case, is high (*blue* line in Fig. 6b.1), since the base length of wrinkle is moderately short as compared to the height of the wrinkle. With the increase in diffused water amount, the base length of the wrinkle keeps increasing while the height is almost the same (Figure S8b–d). Thus, *AR* and ${\theta }_{FAW}$ values drop significantly (*cyan curve* in Fig. 6c).

In Phase I, it is observed that for all cases considered, distributed wrinkles evolve into a localized wrinkle. The way it appears is either the intra-attraction between water molecules to make one droplet, and the vdW attraction between the wrinkle and water molecules that “drag” the graphene along with water droplet^62^. Another possibility is the vdW attraction between the upper graphene sheet and graphene substrate, causing that drag. The probability of the vdW attraction between walls of the same wrinkle, during evolution, are slim to none given the wrinkle wavelength. Furthermore, it may be a combination of all these factors, which may be the function of temporal and spatial variables as well. Though the detailed analysis is beyond the scope of this study, in Phase I, we tried to get an insight into the underlying mechanisms into the wrinkling formation and evolution kinetics.

### Phase II: The dynamics of the localized wrinkle due to water evaporation/drying

**Figure 7:**
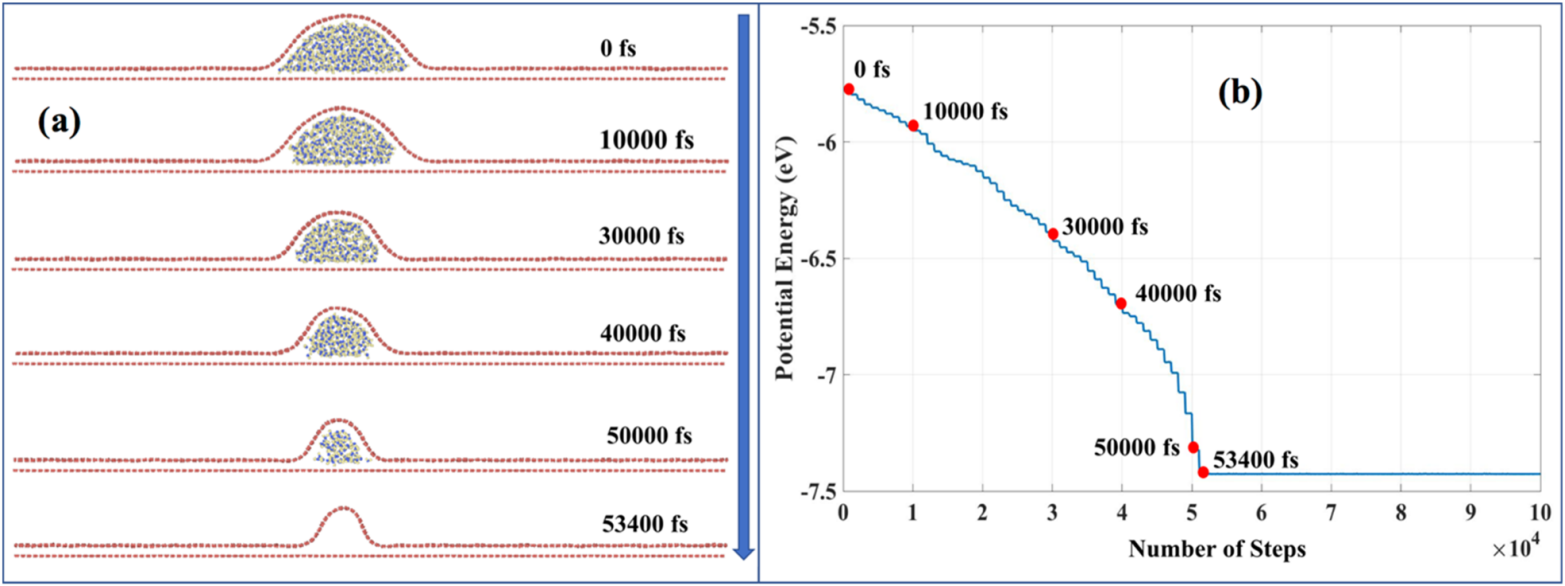
(**a**) The depiction of the water drying process. The system is run by NVE ensemble at 300 K with time step of 1 fs. (**b**) Potential energy variation during the process.

**Figure 8:**
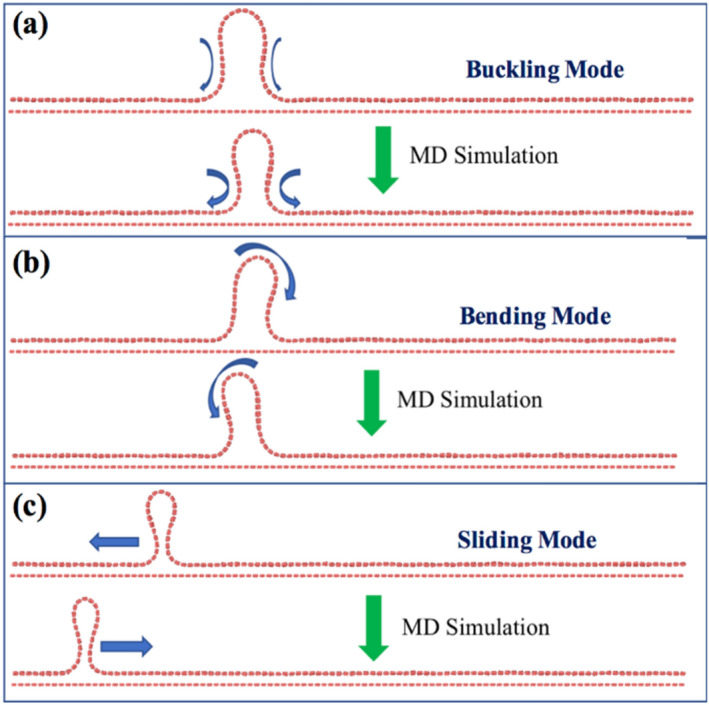
After complete drying, the final localized wrinkled structure can undergo three different modes—(**a**) buckling, (**b**) bending, and (**c**) sliding.

As shown in Fig. 1a, the diffused water evaporates, which changes the structure (*AR* and ${\theta }_{FAW}$) of the localized wrinkle. We consider the final localized wrinkle structure for ${\theta }_{IAW}= 11^\circ$ case with 2-layer water diffusion (Figure S6b) and study the variation in wrinkle configurations upon evaporation. In Fig. 7a, we note the sequence of steps of water evaporation and the change in wrinkle configuration. The SI contains the related video (Movie_Figure7a.mp4). Figure 7b shows the corresponding change in potential energy. We performed the comparative analysis in this plot (as any other potential energy plot in this work), with the least interest in absolute values. When water starts evaporating, the potential energy drops drastically as there is a significant perturbation in the system. Upon drying, there is an approximately 25% decrease in the angle of curvature of wrinkle, which is analogous to the experimental observation shown in Fig. 1. Similarly, we studied the water evaporation and wrinkle formation for other initial angle cases (${\theta }_{IAW}= 6^\circ ,16^\circ ,21^\circ$). Figure S8 shows the wrinkle for 6-layer diffused water case for ${\theta }_{IAW}= 21^\circ$. The SI contains the related video (Movie_FigureS8.mp4). As expected, there was an inverse proportionality in the angle of curvature (Table S3). Here, angle of curvature decreases as opposed to what was observed for other cases, i.e. (${\theta }_{IAW}= 6^\circ ,11^\circ ,16^\circ$). That is attributed to the high slenderness/aspect ratio because of the highest CR among cases considered. We had performed the angle of curvature analysis for all ${\theta }_{IAW}$ considered, as shown in Table S3.

After the complete evaporation, the localized wrinkle undergoes structural changes, which results in three kinds of fundamental movements—(1) *Buckling Mode* (Fig. 8a): The two sides of the wrinkle buckles and reduces the base-length of the wrinkle. However, the height of the wrinkle remains the same (*AR* increases). (2) *Bending Mode* (Fig. 8b): The base length and height of the wrinkle remain the same (*AR* = *constant*). However, the wrinkle bends sideways. (3) *Sliding Mode* (Fig. 8c): In this case, after the complete evaporation, the final localized wrinkle does not remain static at a particular location. Instead, it keeps moving left or right. The sliding mode is more or less present in all cases beyond ${\theta }_{IAW}= 6^\circ$, i.e., after complete evaporation, wrinkle moves sideways. The bending and buckling modes dominate as the initial angle increases. Figure S9 of SI shows the evaporation for a 6-layer water diffusion case. Soon after the complete drying, we notice the buckling at the side of wrinkle. The buckling mode is accompanied by sliding and bending mode (See the related video in SI—Movie_FigureS8.mp4). The bending mode is less dominant for low ${\theta }_{IAW}$ cases ($6^\circ , 11^\circ$) when the height of the wrinkle is lesser.

**Figure 9:**
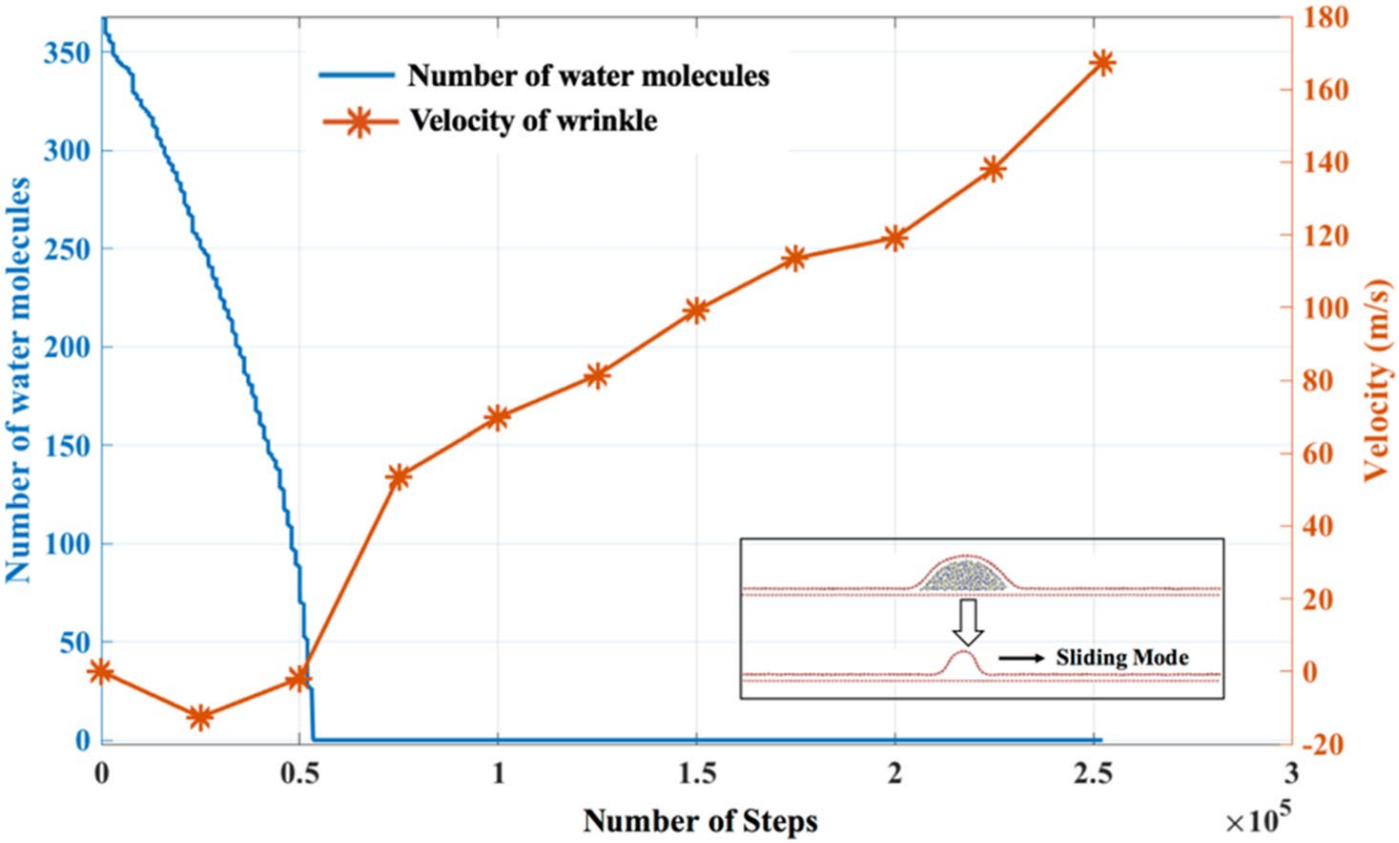
Number of water molecules during the drying process and velocity of the wrinkle (averaged over 50,000 steps).

A critical study is needed to analyze which mode (sliding, bending, buckling) is more dominant at different stages of evaporation, given various initial angles, to tune the wrinkle according to the needs as targeted by other methods^63^. We attempted to get insights into the domination of sliding mode during drying. As mentioned before, for a lower initial angle (e.g., ${\theta }_{IAW}= 11^\circ$), the sliding mode is more prominent. Figure 9 shows the velocity of the wrinkle as a function of water content. The ‘wrinkle-velocity’ is computed with reference to the atom present at the crest of the wrinkle. The identity of the atom located at the crest is changing as wrinkle is moving under the sliding mode. We utilized the ‘in-built’ python-scripting facility in visualization package OVITO^64^ to identify the atomic position at the crest for a different time frame and monitor its movement to compute the velocity. In Figure S10 of the SI, we plotted the number of water molecules present and the corresponding ‘wrinkle-velocity’. Because of the atomic scale movement, there is a lot of noise in the velocity data. Here, positive data represents velocity when wrinkle moves towards the positive *x*-axis, while negative represents when wrinkle moves along the negative *x*-axis. In Fig. 9, the velocity is averaged over every 50,000 steps. We note that as long as water molecules are diffused inside the wrinkle, the speed ranges around zero (until 0.5 × 1*e*5 MD steps). However, once the water molecules are obliterated, then not only velocity jumps to a higher value of magnitude, but it also shows a

positive gradient with MD time steps, and the wrinkle prefers to move towards the positive *x*-axis. This ‘wrinkle-velocity’ pattern indicates that the water molecules diffused under the wrinkle behaves as ‘wrinkle-anchor’ (speed breaker). After the water molecules are completely evaporated, the sideways movement of the wrinkle dominates because the ‘wrinkle-anchor’ is removed. We hypothesize that the direction of the wrinkle movement after the complete evaporation is decided by the complex interplay of three different modes at the moment when the “last-set” of water-molecules is evaporated. However, further critical analysis is needed to validate this proposition.

**Figure 10:**
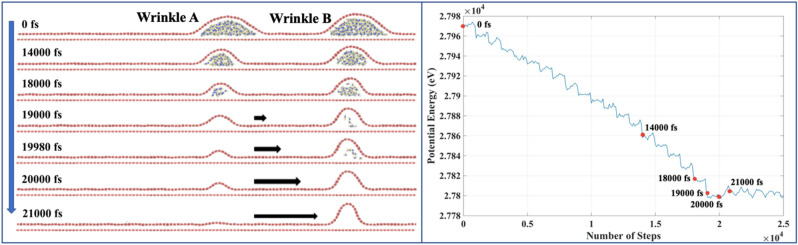
(**a**) Wrinkle amalgamation with water content. The system is run with an NVE ensemble, time step of 1 fs at 300 K. (**b**) Variation of the potential energy during the wrinkling amalgamation.

To understand further the ‘wrinkle-anchor’ phenomenon by the diffused water molecules inside the wrinkle, we considered another set-up, as shown in Fig. 10. We artificially created a scenario where two wrinkles are placed apart, and the different number of water molecules are diffused inside. Different snap-shots are taken until the system has reached the local minima. SI contains the related video (Movie_Figure10a.mp4). Wrinkle-A and Wrinkle-B do not amalgamate until one of them lost all the water molecules diffused underneath. As shown in Fig. 10, until time = 18,000 fs, the vdW interaction between the diffused water and the substrate acts as an ‘anchor support’ to provide the spatial rigidity to the wrinkle. At time = 19,000 fs, Wrinkle-A has the complete evaporation, while for Wrinkle-B, some diffused water molecules are still present. The diffused water molecules act as ‘wrinkle-anchor’ (speed breaker) for Wrinkle-B. Moreover, the presence of water molecules inside Wrinkle-B also prevents the sliding mode of Wrinkle-A. In fact, instead of sliding mode, here Wrinkle-A starts collapsing after complete evaporation since Wrinkle-B prevents its sideway motion. After complete evaporation for Wrinkle-B (time = 20,000 fs), Wrinkle-A collapses completely and merges with Wrinkle-B. Therefore, unlike single wrinkle cases (Fig. 7), when two wrinkles are present, the motion of one wrinkle not only depends on the amount of water diffused underneath it but also on the water underneath the adjacent wrinkle. We hypothesize that in a network of wrinkles in a 2D material, we can control the evolution of the wrinkle-network by controlling the amount of water content trapped under fewer wrinkles than the total number of wrinkles participating in the wrinkle-network. It will be valuable observation^1^ if we can control the evolution of the whole wrinkle-network by controlling the water content trapped under just one wrinkle, which may be or may not be the member of the original wrinkle-network.

### Phase III: DFT study of the electronic properties of winkled graphene

#### Crystal orbitals (HOMO and LUMO)

**Figure 11:**
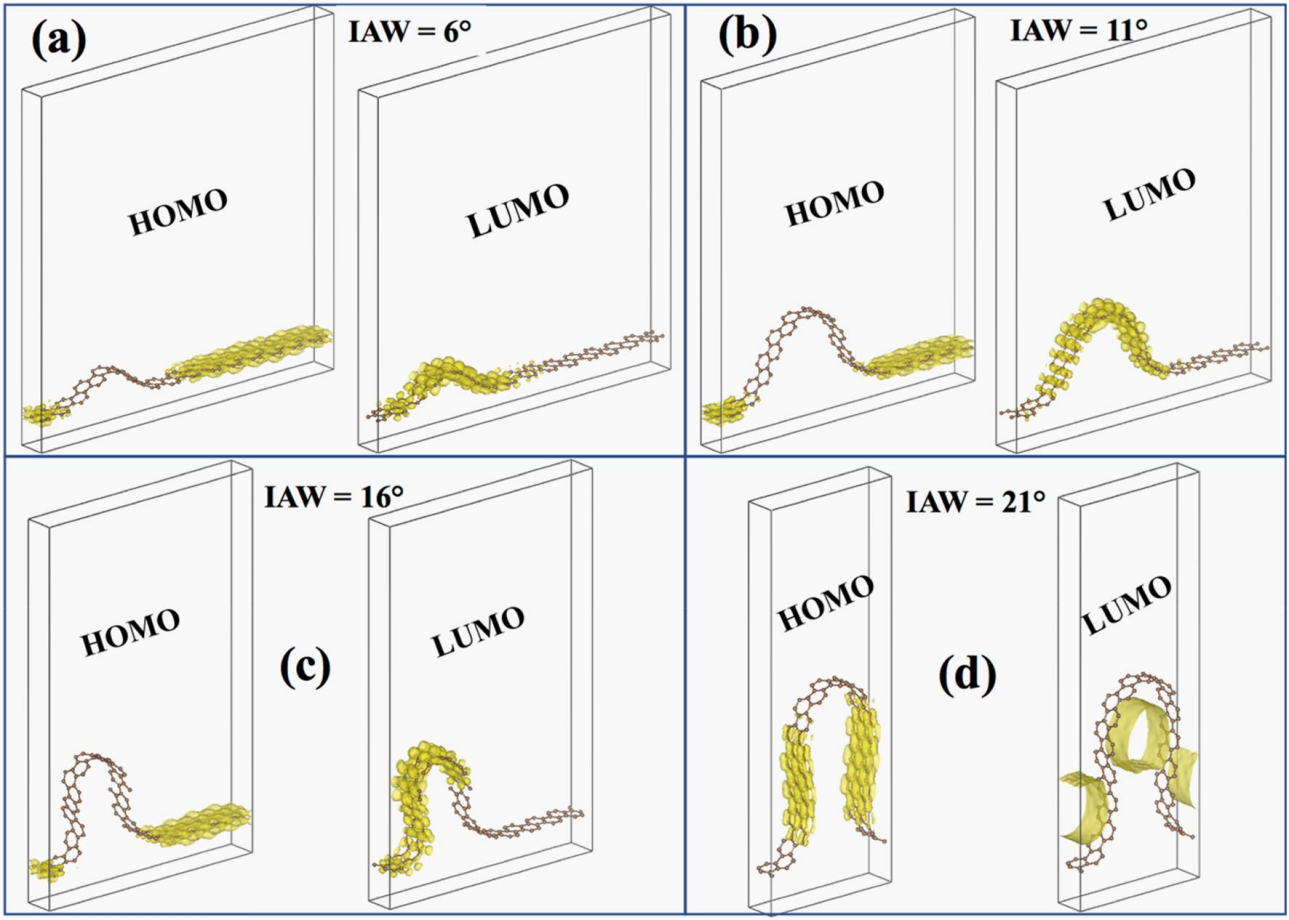
Isometric view of HOMO and LUMO for four localized wrinkle obtained after complete evaporation of wrinkled graphene (four water-layer case) with Initial Angle of Wrinkle (IAW) of (**a**) 6°, (**b**) 11°, (**c**) 16°, and (**d**) 21°.

We performed the Molecular Orbital (MO) analysis of WGNR for all four angles ($6^\circ ,11^\circ ,16^\circ ,21^\circ$). We considered the localized wrinkle structure for four-layer of water cases shown in Figures S5c, S6c, S7c and S8c. Then we performed the complete evaporation. The resulting localized wrinkle after complete evaporation is considered here, excluding the underlying substrate, i.e., free-standing WGNR (Fig. 11). We considered the same number of atoms in all the cases, i.e., 120 atoms. The system is optimized with the MD code before performing electronic calculations with DFT. Figure 11 shows the isometric view and Fig. S12 of SI shows the front view. Crystal orbitals are demonstrated by the iso-surfaces with yellow color. A carbon atom has six electrons, out of which two lowest electrons are considered as core electrons, and the next four electrons are regarded as valence electrons. These valence electrons participate in the interaction with other atoms in the crystal. Consequently, we are considering these four electrons in the outmost shell in the DFT calculations.

We observed the Highest Occupied Molecular Orbital (HOMO) at the portion of the WGNR, where the the radius of curvature is high, i.e., the flat portion (Fig. 11). For IAW of 6°, 11°, & 16°, HOMO is observed to be only on the flat part of the GNR. This indicates that lattice distortion, present due to the wrinkle, is increasing the energy level of the molecular orbitals forming up the wrinkled portion of GNR. Hence, the electrons prefer to fill up the molecular orbitals formed by the flat portion of the GNR. The same phenomenon is observed in the sidewalls of the wrinkle for the highest angle considered (21°). In this case, the wrinkle is slender enough so that its sidewalls act like flat graphene and form the molecular orbitals having less energy. Consequently, electrons prefer to stay in the molecular orbitals formed by the sidewalls of the wrinkle instead of the vertex.

The Lowest Unoccupied Molecular Orbital (LUMO) is mostly observed in the portion of the GNR having high curvature (Fig. 11). From IAW of 6° to 16°, LUMO can be found in the wrinkled section of the GNR but definitely not in the flat portion. It indicates that if the GNR gains an electron, then it will be “absorbed” by the atoms forming the wrinkle. The effect of curvature plays a dominant role when we increase the IAW beyond 16°. In the case of 21°, we observe LUMO in the form of tunnel shape^65^. The wrinkle is slender enough to confine the lowest unoccupied molecular orbital parallel to its direction. Further investigation is necessary to understand whether it’s just the effect of curvature or the presence of HOMO along the sidewalls of the wrinkle or both of these factors contributing to some extent to the formation of “electron-tunnel”-LUMO. This observation proves that not only the presence of wrinkles but its slenderness also plays a crucial role in the generation of “tunnel” LUMO orbital. It can be utilized to control the direction of electron flow in a (semi)conducting system, as observed by Sulpizio et al. in the form of electron flow following a macro hydrodynamic flow pattern, i.e., Poiseuille flow^66^.

Similar to these ‘electron-tunnels’, ‘electron-channels’ are obtained in the IAW values of 6°, 11°, 16°. The ‘electron-channels’ is the overlapping of the atomic orbitals of the same energy level forming an “electron-flow-channel” along the direction of wrinkle, which can act as a tunnel to the flow of every extra electron added to the system. Consequently, by controlling the course of these channels, the direction of electron-flow can also be controlled, which has multiple applications in the semiconductor industries. The studies aimed for similar outcomes are available in the literature^66,67^. For IAW of 16°, the asymmetricity is observed for LUMO. As shown in Fig. 11c (SI Figure S12c), the wrinkle is bent on one side (left). That causes the LUMO to only form on the curved sidewall completely and partially on the right sidewall. There is a portion of the right sidewall of the wrinkle, where no LUMO or HOMO is observed (SI Figure S12c). Further investigation is necessary to determine the energy level of the orbitals, possessed by the corresponding set of atoms forming the portion of the right sidewall of the wrinkle, where neither LUMO nor HOMO is obtained.

#### Band structure

**Figure 12:**
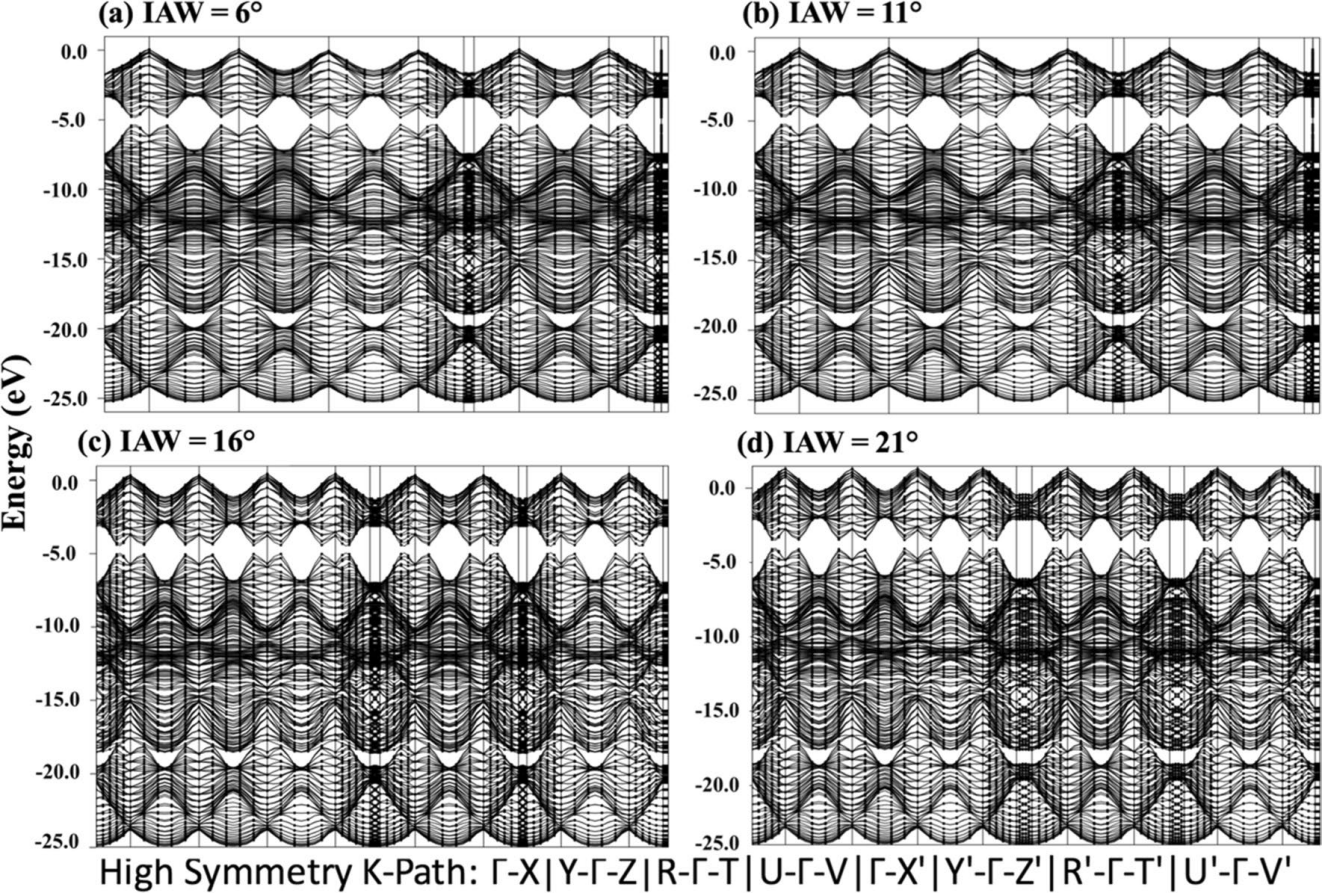
Band structure along high symmetry k-points for four localized wrinkle obtained after complete evaporation of wrinkled graphene (four water-layer case) with Initial Angle of Wrinkle (IAW) of (**a**) 6°, (**b**) 11°, (**c**) 16°, and (**d**) 21°.

In Fig. 12, band structures are plotted for localized wrinkled graphene structures shown in Fig. 11. Each band structure is based upon the system consisting of 240 bands corresponding to 480 electrons constituting the crystal. The direct bandgap is observed for all of the cases. The observed bandgap values, based upon the difference between HOMO and LUMO, are 0.5492, 0.5453, 0.4801, and 0.5266 eV for the localized wrinkled structure corresponding to the IAW cases of 6°, 11°, 16°, 21°, respectively. Figure S11 in SI shows the band structure of the flat pristine graphene. As expected, no bandgap is observed for this case.

The bandgap is observed to be decreasing linearly with IAW (6°, 11°, 16°), but then it increases suddenly for 21°. This can be attributed to the observation made in Fig. 11d for the 21° case, i.e., distortion of the HOMO–LUMO. As explained before, because of slenderness, the HOMO is observed on the sidewalls of the wrinkle. Moreover, because of high curvature, LUMO shifted from the tip to under the wrinkle to form an “electron tunnel”. The delocalization of the HOMO and LUMO orbital for the highest angle case (21°) may be the reason behind non-linearity in the proportional relationship of IAW and bandgap.

#### Density of states (DOS)

**Figure 13:**
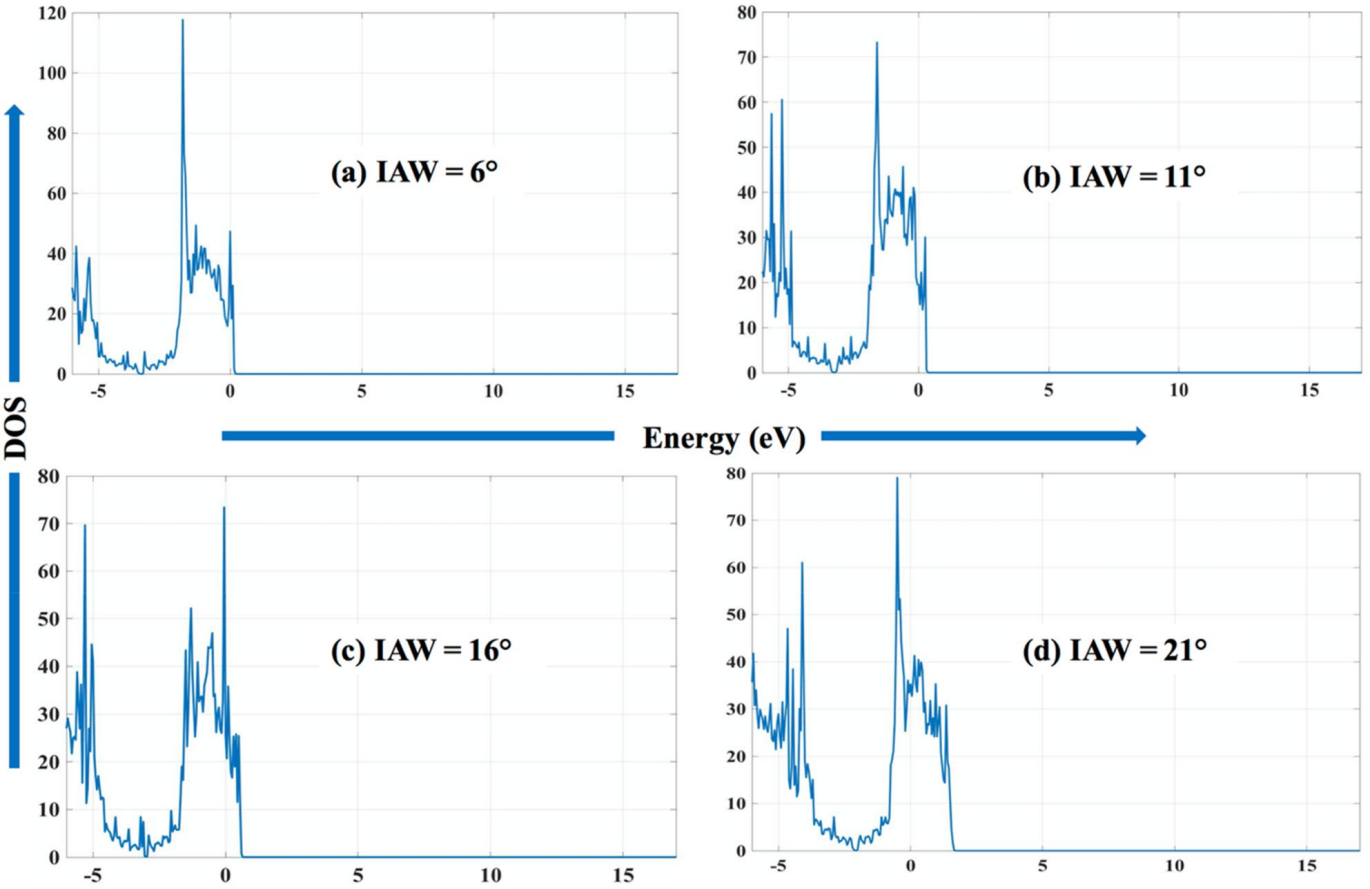
Density of states for four localized wrinkle obtained after complete evaporation of wrinkled graphene (four water-layer case) with Initial Angle of Wrinkle (IAW) of (**a**) 6°, (**b**) 11°, (**c**) 16°, and (**d**) 21°.

In Fig. 13, the density of states (DOS) is plotted for four wrinkled graphene structures mentioned in Fig. 11. An inversely proportional relationship is observed between Fermi energy and the IAW. As IAW increases (6°, 11°, 16°, 21°), the magnitude of the Fermi energy decreases as − 3.3651 eV, − 3.2326 eV, − 2.9825 eV, − 2.0637 eV, respectively. The Fermi energy obtained for flat graphene is − 2.3462 eV, which agrees with the values reported in the literature^68^. With the increase in IAW, the angle of curvature of final localized wrinkle decreases. It means that the wrinkled portion of GNR is denser (number of atoms/volume) than the flat portion of GNR and hence more stretched bonds.

Consequently, the strained lattice increases the energy levels of the localized crystal orbitals, which results in changing the Fermi energy as a function of IAW. In Fig. 13, we notice two prominent peaks in DOS plots for all cases (IAW = 11°, 16°, 21°) except for the least IAW case (6°). We have the second peak in all the cases at around − 2 eV to 0 eV, and the first peak at around − 5 eV except for the least IAW case (6°). Further work can be conducted to find out the first value of IAW, which will generate the first peak in between IAW of 6° to 11° and results in two peaks.

As can be seen from potential energy plots (Figure S1−S4), the local minima of potential energy increase (− 7.427 eV < − 7.426 eV  < − 7.424 eV < − 7.423 eV) as the initial angle of wrinkle increases (6° < 11° < 16° < 21°). And the corresponding relationship can be observed in band structure plots while observing the Valence Band Maximum (VBM) in Fig. 12. As the initial angle of the wrinkle is increased, so does the VBM from approximately 0–1.5 eV. Synchronously, in total DOS plot in Fig. 13, the energy corresponding to the highest energy state is also increased from ≈0 eV to ≈2 eV.

## Conclusions

In conclusion, we have performed MD and DFT simulations to geometrically and electronically characterize the wrinkle formation, evolution, and collapse in graphene when water is diffused in between graphene and substrate, and while it is evaporating/had evaporated. Our key findings are summarized below:

- The distributed wrinkles in graphene coalesce to form a ‘localized wrinkle’, whose configuration significantly depends on the initial pattern of distributed wrinkles and the amount of diffused water present in between the graphene and substrate. Upon evaporation of diffused water, the localized wrinkles, except with the highest initial angle, lose their angle of curvature values in the range from 7 to 63%.
- When water is distributed on a graphene sheet, due to hydrophobicity, distributed water molecules condense to form a water droplet. In the presence of diffused water, the final wrinkle configuration is the result of the competition between the water droplet formation process and the localized wrinkle formation process. For the lower initial angle of wrinkle, droplet formation is dominant, resulting in wrinkle with a high angle of curvature values, i.e., non-sharp vertex. For higher initial wrinkle angle, wrinkle formation dominates, and the resulted localized wrinkle tends to possess a lower angle of curvature, i.e., sharp vertex.
- The drying of water, diffused under the localized wrinkle, changes its configuration, e.g., curvature/vertex angle. The wrinkle is static until the complete evaporation, and after that, it acquires a motion. In case of diffused water under two localized wrinkles placed side-by-side, even after the complete evaporation under one wrinkle, it remains static until the water diffused under the neighboring wrinkle is completely dried.
- The wrinkle movement is the combination of three fundamental modes—bending, buckling, and sliding. The dominant mode depends on the configuration of the wrinkle., e.g., relatively narrowed or wrinkles with relatively a lower angle of curvature tends to bend. Further investigation is necessary to understand how three different modes influence wrinkle dynamics.
- The stress analysis reveals that the maximum stress is at the base of the wrinkle, and always below its plasticity limit. Therefore, wrinkle formation due to water diffusion does not cause any significant bond-breaking.
- “Electron-tunnel/channel” is observed along the vertex of the WGNR. By controlling the direction of WGNR, we can control the direction of the electron flow. This concept can be used in designing flexible electronics and optoelectronics.
- The direct bandgap is observed for all the wrinkle cases considered. The change in wrinkle geometry also generates lattice strains, which further changes the energy level of the crystal orbitals. The strain in lattice changes the Fermi energies which results in Fermi level modulations.

Our study addresses the fundamental questions regarding the wrinkle formation, evolution, and collapse due to water diffusion/evaporation, and its importance in flexible electronics.

## Supplementary information

Supplementary file1

Movie_Figure4a.mp4

Movie_Figure4b.mp4

Movie_Figure4c.mp4

Movie_Figure7a.mp4

Movie_Figure10a.mp4

Movie_FigureS8.mp4

## Data Availability

The data reported in this paper is available from the corresponding author upon reasonable request.

## Abbreviations

FG: Flat graphene
GDW: Graphene with distributed wrinkles
GLW: Graphene with localized wrinkle
GNR: Graphene nano-ribbons
WGNR: Wrinkled graphene nanoribbons
IAW: Initial angle of wrinkling (${\uptheta }_{\mathrm{I}\mathrm{A}\mathrm{W}}$)
FAW: Final angle of wrinkle (${\uptheta }_{\mathrm{F}\mathrm{A}\mathrm{W}}$)
PBC: Periodic boundary conditions
CR: Carbon ratio
AR: Aspect ratio

## Code availability

The pre- and post-processing codes used in this paper are available from the corresponding author upon reasonable request. Restrictions apply to the availability of the simulation codes, which were used under license for this study.
