# Supplementary material for: Computational study of the water-driven graphene wrinkle life-cycle towards applications in flexible electronics
Source: Sci Rep. 2020 Jul 9;10:11315. doi: 10.1038/s41598-020-68080-5 (PMC7347945; doi:10.1038/s41598-020-68080-5)
Supplement: Supplementary file 1 — Supplementary file1 [file 41598_2020_68080_MOESM1_ESM.docx]

**SUPPLEMENTARY INFORMATION**

**Computational study of the water-driven graphene wrinkle life-cycle towards applications in flexible electronics**

Jatin Kashyap^1^, Eui-Hyeok Yang^2^, Dibakar Datta^*,1^

^1^ Department of Mechanical and Industrial Engineering, New Jersey Institute of Technology, Newark, NJ 07103, USA

^2^ Department of Mechanical Engineering, Stevens Institute of Technology, Hoboken, NJ 07030, USA

*Corresponding Author (Email: dibakar.datta@njit.edu)

1. **Tables**

**Table S1:** Number of carbon atoms in the upper and lower graphene for different $\theta_{IAW}$

|  | $\theta_{IAW}=$6$^{\circ}$ | $\theta_{IAW}=$11$^{\circ}$ | $\theta_{IAW}=$16$^{\circ}$ | $\theta_{IAW}=$21$^{\circ}$ |
| --- | --- | --- | --- | --- |
| Number of C in the **Upper** ‘Graphene  with Wrinkles’ | 1920 | 1920 | 1920 | 1920 |
| Number of C in **Lower** Flat Graphene | 1888 | 1824 | 1728 | 1600 |
| ***Carbon Atom Ratio (CAR)*** between the Upper Graphene & Lower Graphene | 1.0169 | 1.0526 | 1.1111 | 1.2000 |

**Table S2:** Number of water molecules for different cases

| Water Layer | $\theta_{IAW}=$6$^{\circ}$ | $\theta_{IAW}=$11$^{\circ}$ | $\theta_{IAW}=$16$^{\circ}$ | $\theta_{IAW}=$21$^{\circ}$ |
| --- | --- | --- | --- | --- |
| 0 | 0 | 0 | 0 | 0 |
| 2 | 384 | 368 | 352 | 320 |
| 4 | 768 | 736 | 704 | 640 |
| 6 | 1152 | 1104 | 1056 | 960 |

Table S3: Angle of curvature measurements for 2-layer cases

| IAW ($\theta_{IAW}$) | Initial angle | Final angle | % Change |
| --- | --- | --- | --- |
| 6° | 24.0365° | 39.1605° | 62.92 |
| 11° | 50.4876° | 63.4349° | 25.64 |
| 16° | 70.2189° | 75.5792° | 7.63 |
| 21° | 93.1162° | 97.8855° | -5.12 |

Table S4: van der Waals contributions for 2-layers cases

| Angle  ($\theta_{IAW}$) | van der Waals contribution(eV), before evaporation | van der Waals contribution(eV), after evaporation |
| --- | --- | --- |
| 6° | -28225.702 | -28285.207 |
| 11° | -27746.977 | -27803.604 |
| 16° | -27052.385 | -27085.429 |
| 21° | -26086.642 | -26129.623 |

1. **Figures**


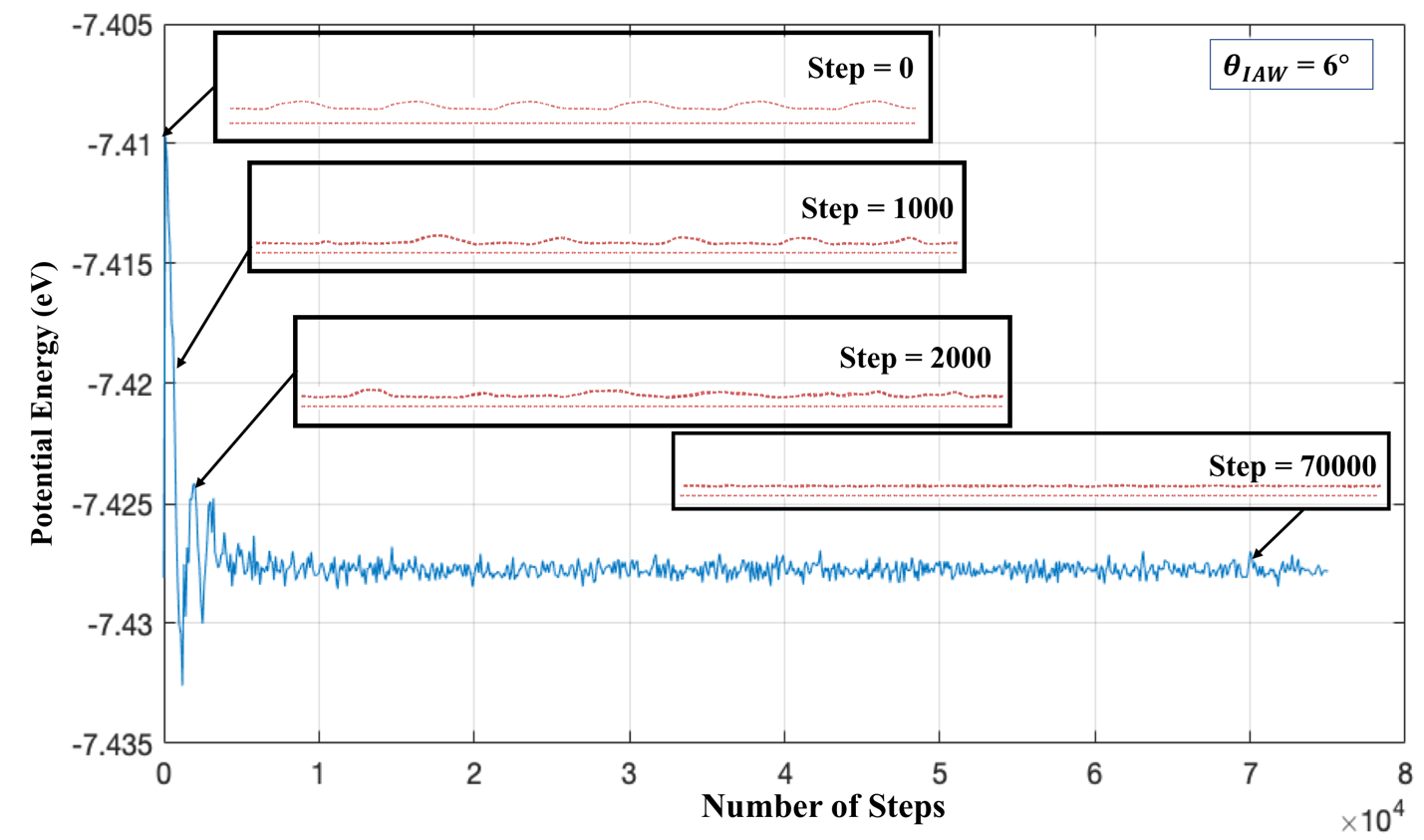


**Figure S1**: Potential Energy variation for initial wrinkle angle of $6^{\circ}$(without water case).


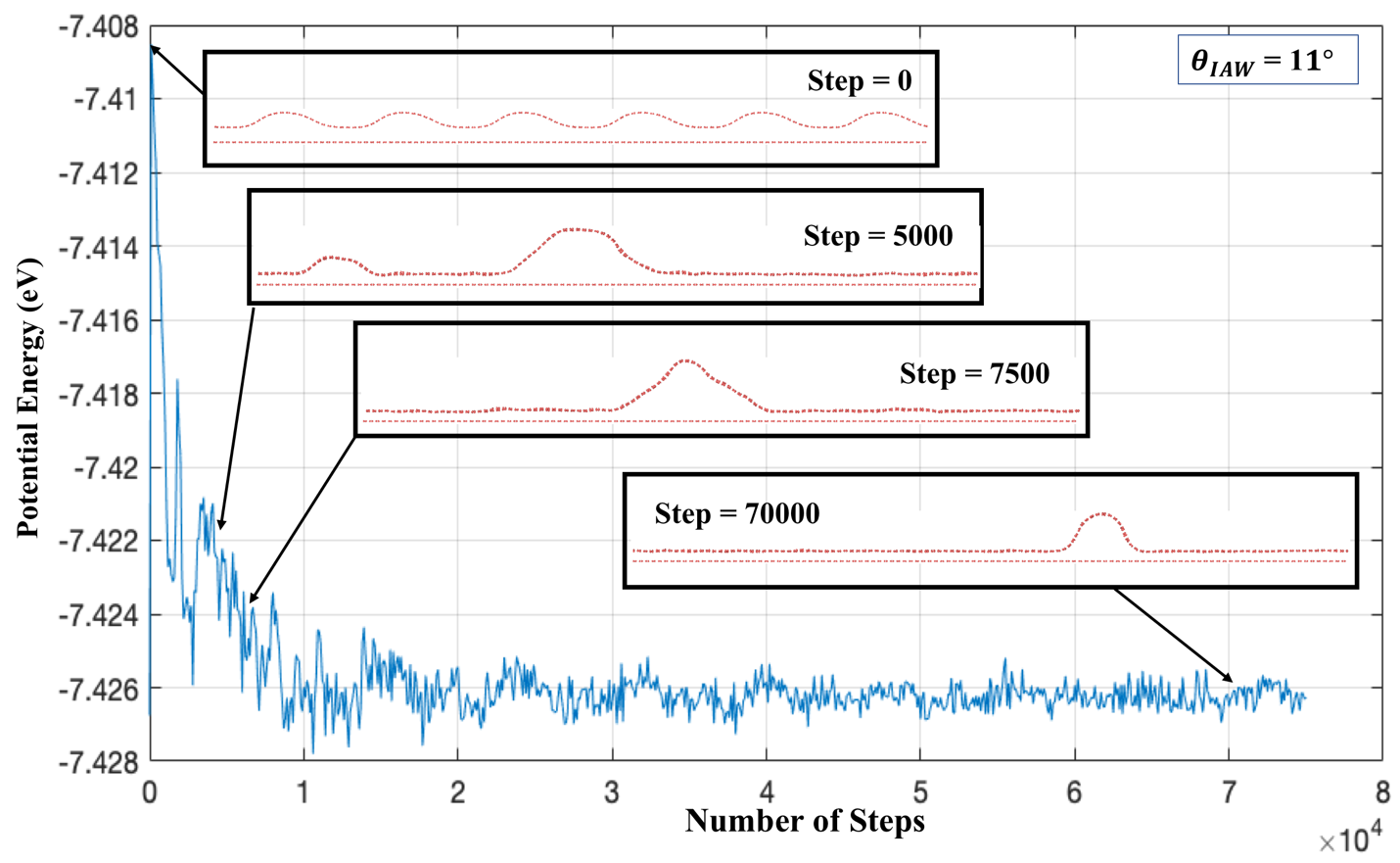


**Figure S2:** Potential Energy variation for initial wrinkle angle of $11^{\circ}$(without water case).


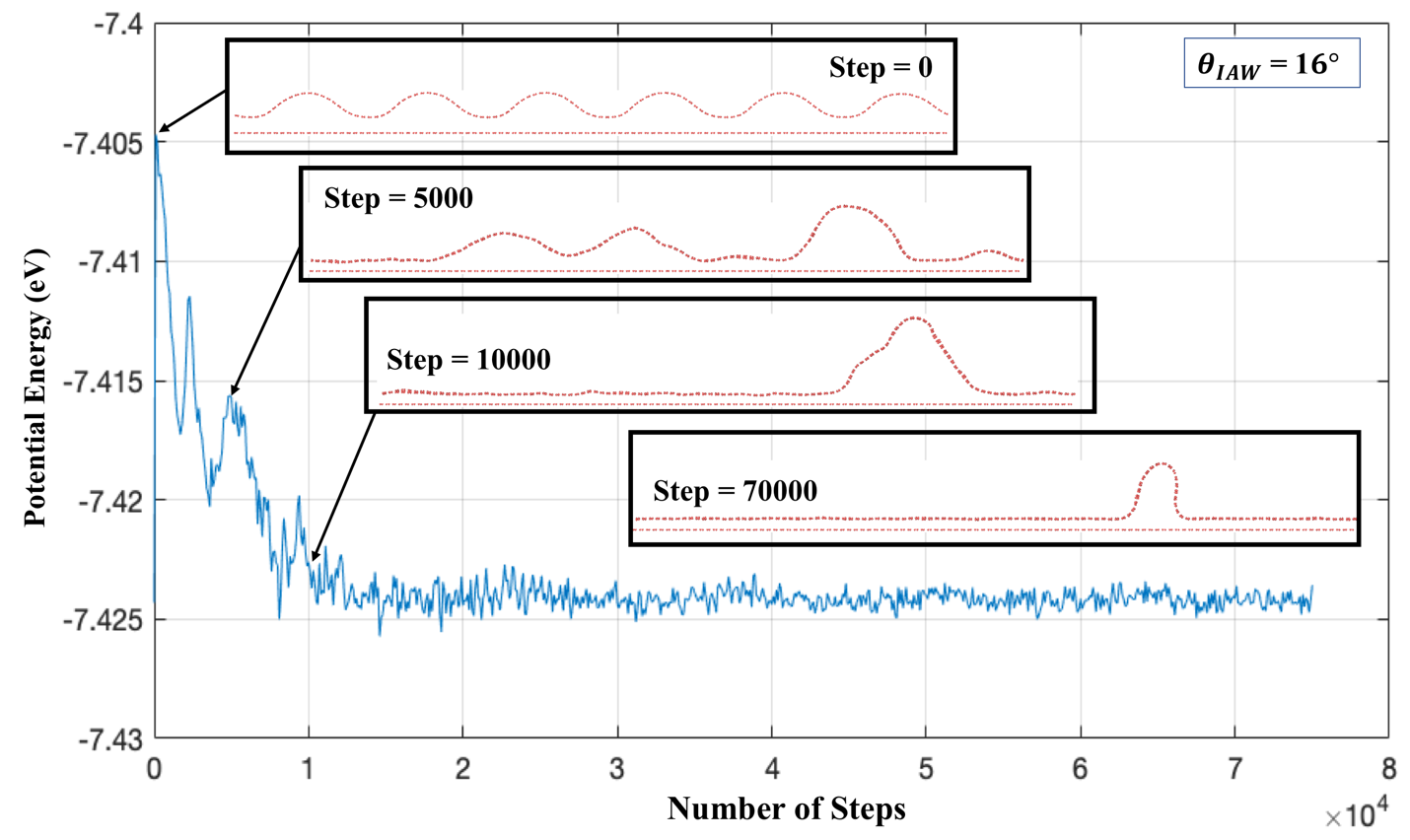


**Figure S3:** Potential Energy variation for initial wrinkle angle of $16^{\circ}$(without water case).

**
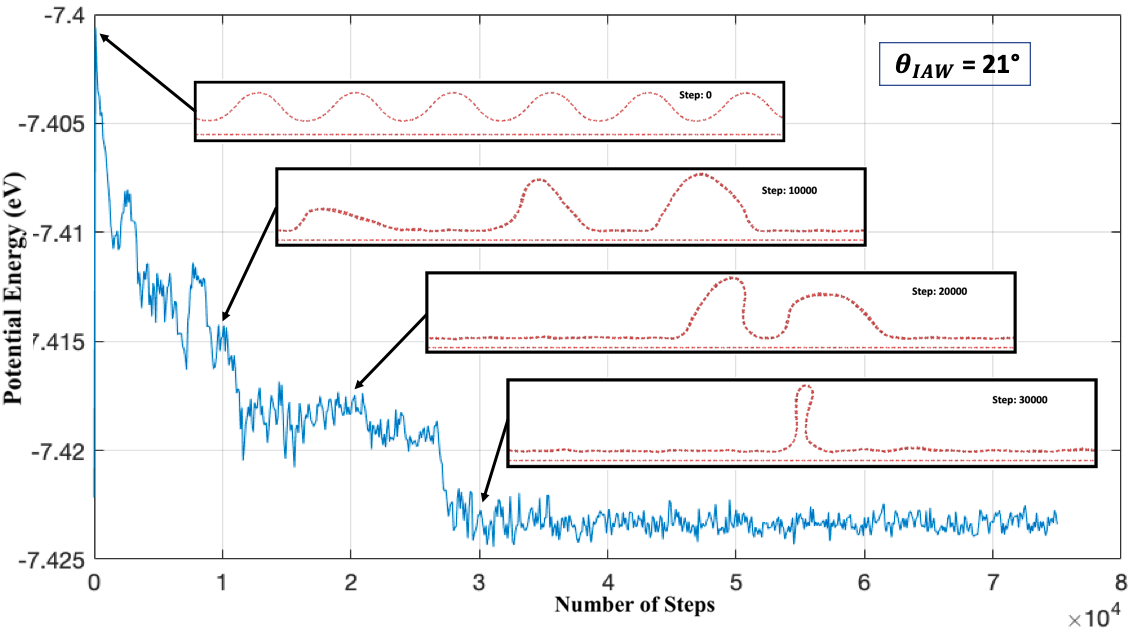
**

**Figure S4:** Potential Energy variation for initial wrinkle angle of $16^{\circ}$(without water case).

**Figure S5:** Final equilibrium structure with stress profile starting from initial wrinkle angle of 6$^{\circ}.$


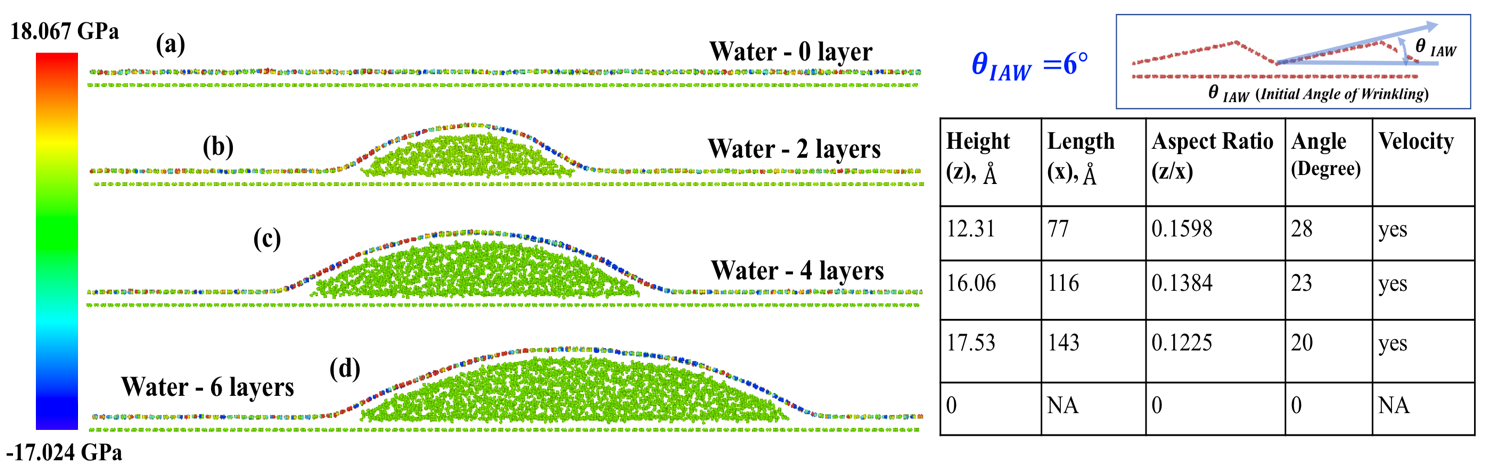


**Figure S6:** Final equilibrium structure with stress profile starting from initial wrinkle angle of 1$1^{\circ}.$


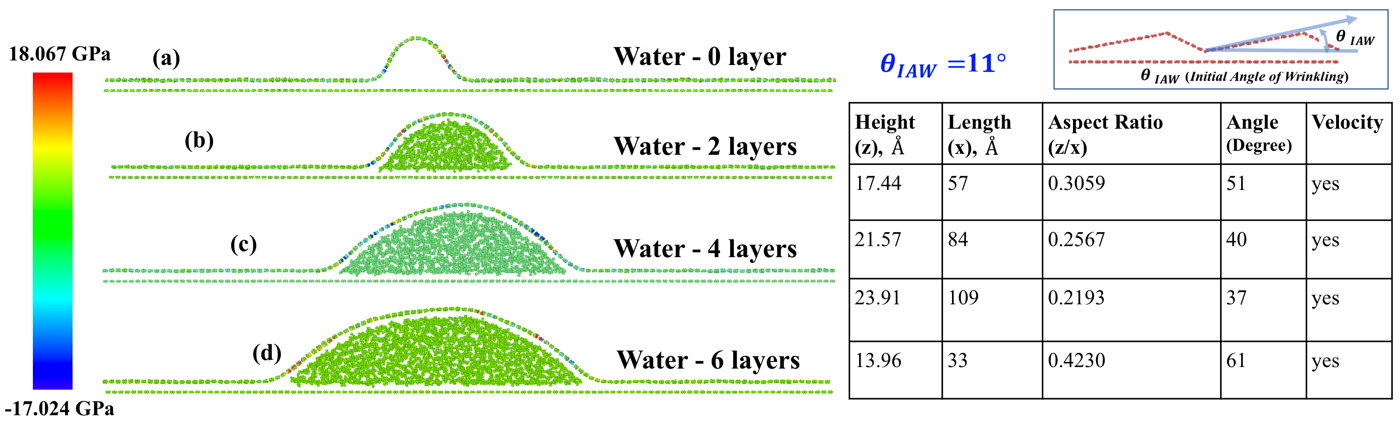


**Figure S7:** Final equilibrium structure with stress profile starting from initial wrinkle angle of 16$^{\circ}.$


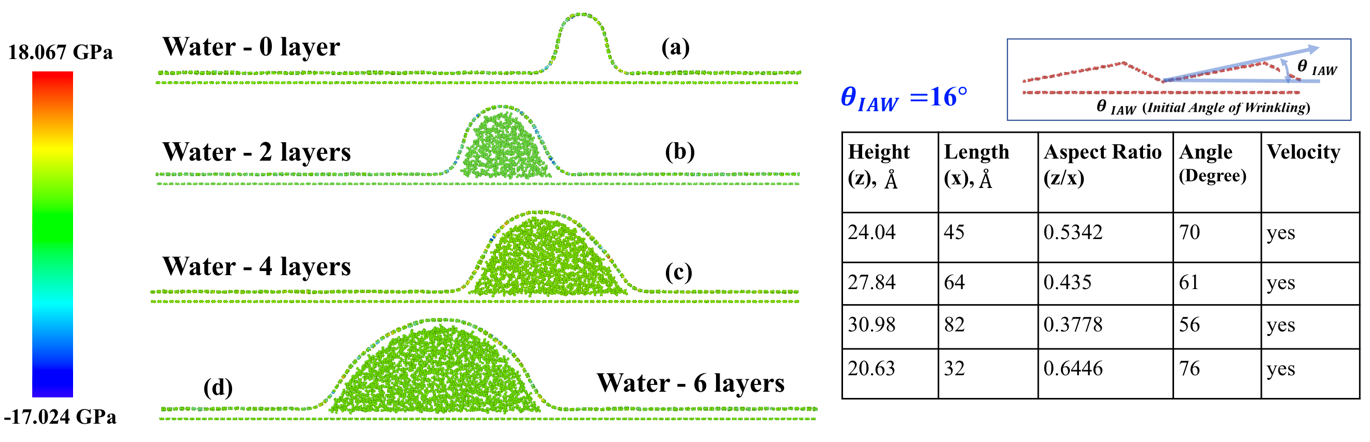


**Figure S8:** Final equilibrium structure with stress profile starting from initial wrinkle angle of 21$^{\circ}.$


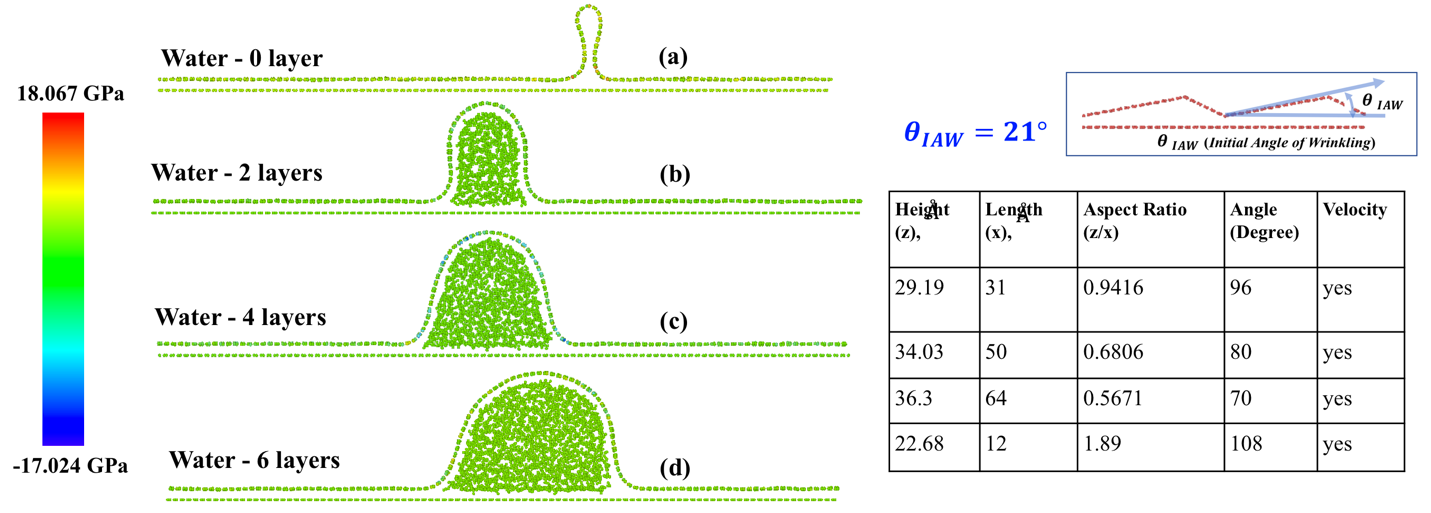


**Figure S9:** Final equilibrium structure starting from initial wrinkle angle of 2$1^{\circ}.$


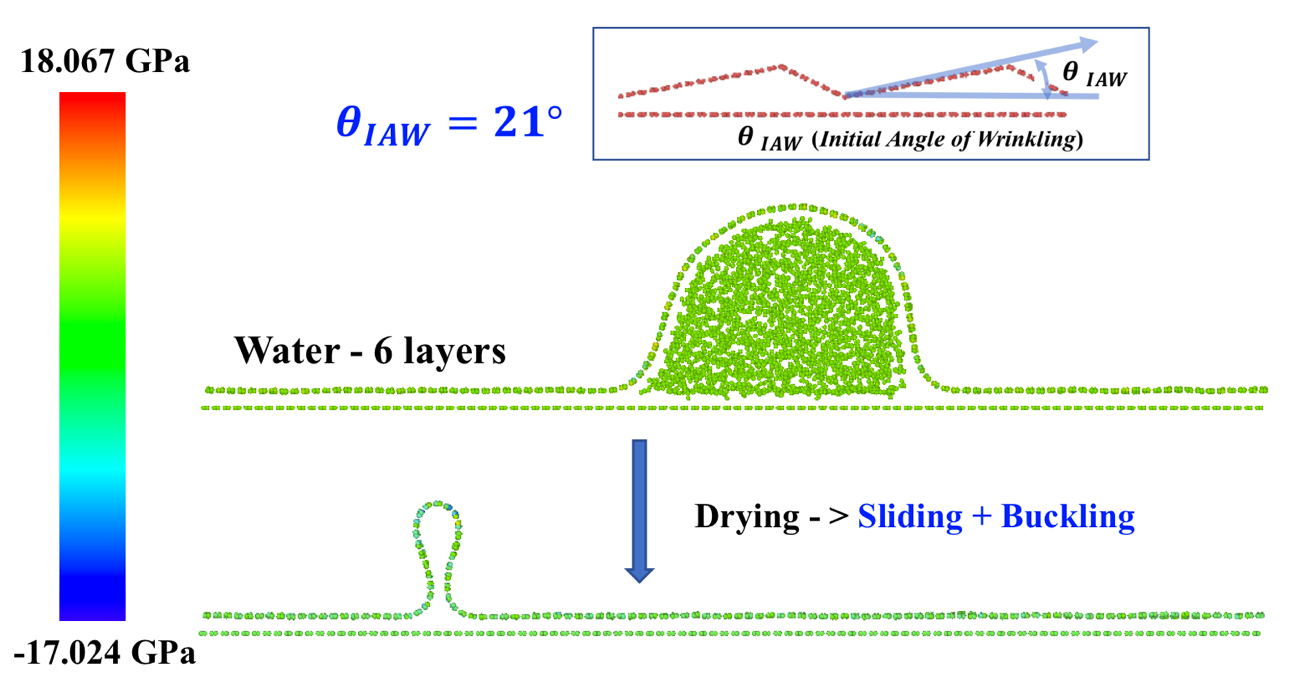

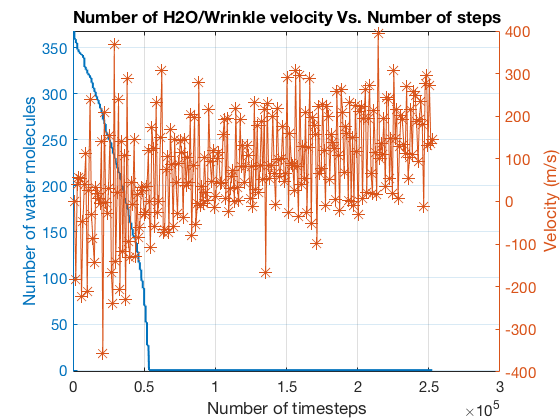


**Figure S10:** Velocity of the wrinkle and the number of water molecules due to evaporation. Here, instantaneous velocity is considered.


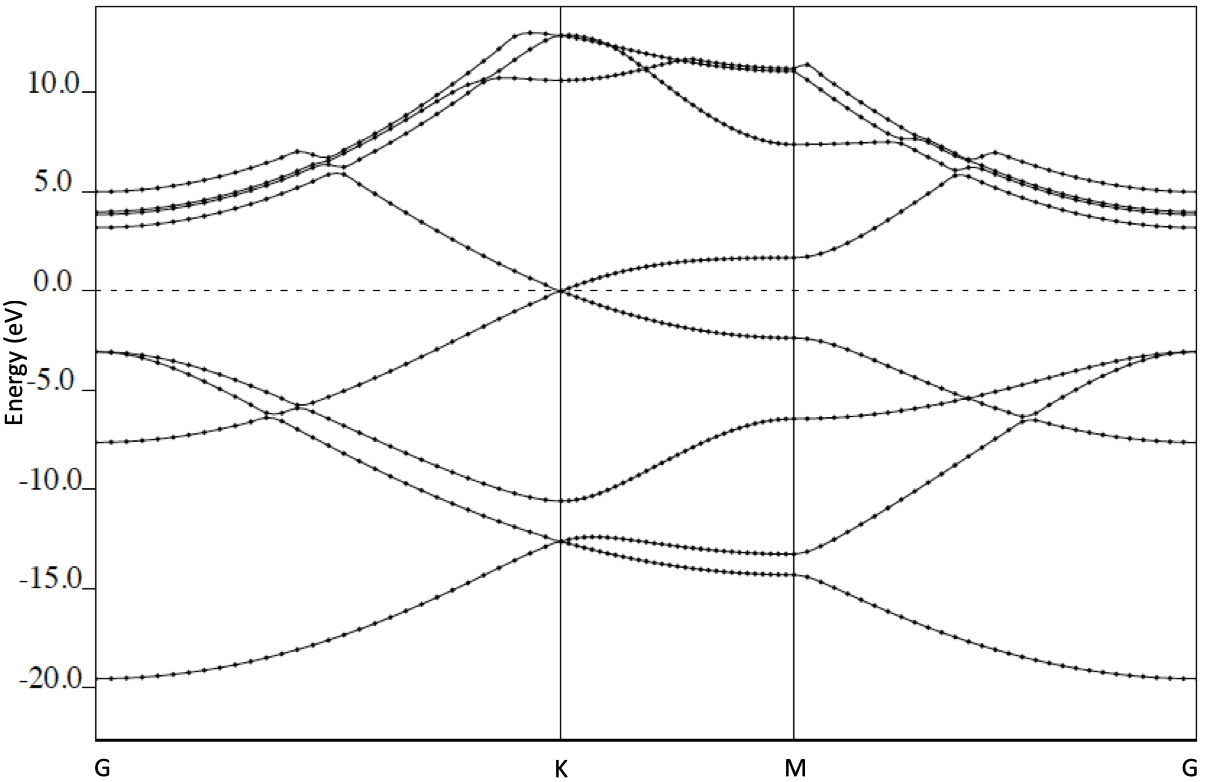


**Figure S11:** Band structure of pristine flat graphene.


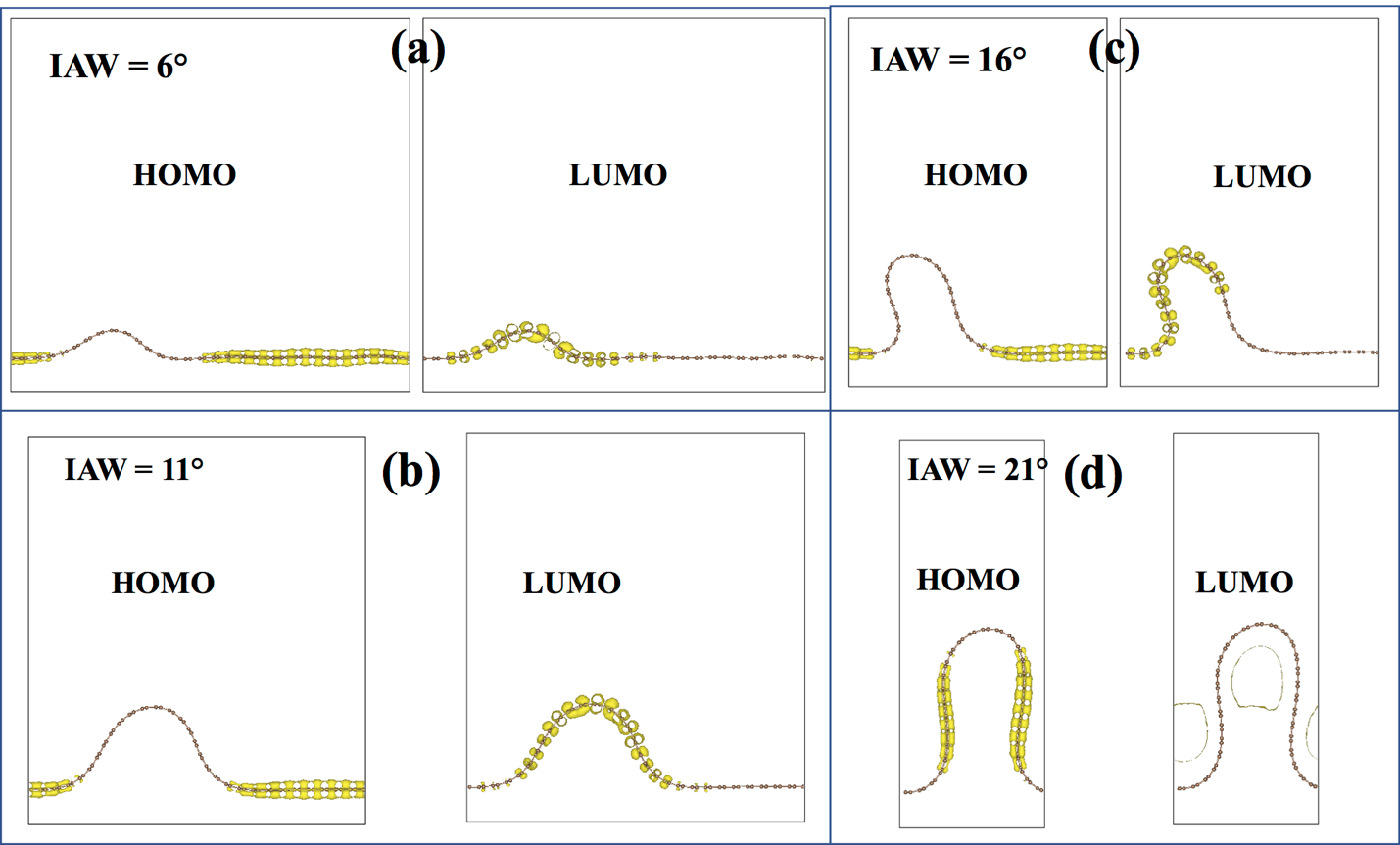


**Figure S12**: Front view of HOMO and LUMO for four localized wrinkle obtained after complete evaporation of wrinkled graphene (four water-layer case) with Initial Angle of Wrinkle (IAW) of (a) 6°, (b) 11°, (c) 16°, and (d) 21°.

1. **Movie Files**

| **Sl. No.** | **Movie File Name** | **Corresponding Figure File Name** |
| --- | --- | --- |
| 1 | Movie_Figure4a.mp4 | Figure4a |
| 2 | Movie_Figure4b.mp4 | Figure4b |
| 3 | Movie_Figure4c.mp4 | Figure4c |
| 4 | Movie_Figure7a.mp4 | Figure7a |
| 5 | Movie_Figure10a.mp4 | Figure10a |
| 6 | Movie_FigureS8.mp4 | FigureS8 |

1. **Detail of the stress calculation.**

The stress tensor for atom I is given by Equation 1 [1]. Here, subscripts *a* and *b* are substituted with the values of spatial variables associated with given atom I to produce different values of the stress tensor. The first term is a contribution from kinetic energy of atom I. The second term gives the virial contribution associated with intramolecular and intermolecular interactions as given by Equation 2.

$S_{ab}= -mv_{a}v_{b}-W_{ab}$ (1)

Here, m and v are mass and velocity of the atom I.

In Equation 2, the first term is the pairwise energy contribution of atom I, where *n* sums over *N_p_* neighbor atoms. *F_1_, F_2_* and *r_1_, r_2_* are forces and positions associated with atoms considered in the pairwise interaction, respectively. The second term contributes to the bonds formed with atom I. Here, the summation is performed over atoms bonded with atom I according to the given potential field. The third, fourth and fifth terms are contributing in the same way as the second term, but the only difference is these terms are considering the angle, dihedral, and improper interactions of the atom I, respectively.

$$W_{ab}=\frac{1}{2}\sum_{n=1}^{N_{p}} \left( r_{1_{a}}F_{1_{b}}+r_{2_{a}}F_{2_{b}} \right)+\frac{1}{2}\sum_{n=1}^{N_{b}} \left( r_{1_{a}}F_{1_{b}}+r_{2_{a}}F_{2_{b}} \right)+\frac{1}{3}\sum_{n=1}^{N_{a}} \left( r_{1_{a}}F_{1_{b}}+r_{2_{a}}F_{2_{b}}+r_{3_{a}}F_{3_{b}} \right)+ +\frac{1}{4}\sum_{n=1}^{N_{d}} \left( r_{1_{a}}F_{1_{b}}+r_{2_{a}}F_{2_{b}}+r_{3_{a}}F_{3_{b}}+r_{4_{a}}F_{4_{b}} \right)+ +\frac{1}{4}\sum_{n=1}^{N_{i}} \left( r_{1_{a}}F_{1_{b}}+r_{2_{a}}F_{2_{b}}+r_{3_{a}}F_{3_{b}}+r_{4_{a}}F_{4_{b}} \right)+ Kspace\left( r_{i_{a}},F_{i_{b}} \right)+\sum_{n=1}^{N_{f}} r_{i_{a}}F_{i_{b}}$$

 (2)

The sixth term defines the contribution by long range columbic interactions, which are defined in the phase -II of the study due to absence of charged particles, i.e., water molecules. The last term is giving the contribution by constraints used to restrict the movement of carbon atoms of the substrate, i.e, flat graphene (FG).

**Reference**

[1] A. P. Thompson, S. J. Plimpton, and W. Mattson, “General formulation of pressure and stress tensor for arbitrary many-body interaction potentials under periodic boundary conditions,” *J. Chem. Phys.*, vol. 131, no. 15, p. 154107, Oct. 2009.
